# Phenolome of Asian Agrimony Tea (*Agrimonia asiatica* Juz., Rosaceae): LC-MS Profile, α-Glucosidase Inhibitory Potential and Stability

**DOI:** 10.3390/foods9101348

**Published:** 2020-09-23

**Authors:** Nina I. Kashchenko, Daniil N. Olennikov

**Affiliations:** 1 Laboratory of Medical and Biological Research, Institute of General and Experimental Biology, Siberian Division, Russian Academy of Science, 6 Sakh’yanovoy Street, Ulan-Ude 670047, Russia; olennikovdn@mail.ru; 2 Department of Biology, Institute of Natural Sciences, North-Eastern Federal University, 58 Belinsky Street, Yakutsk 677027, Russia

**Keywords:** *Agrimonia asiatica*, Rosaceae, ellagitannins, agrimoniin, high-performance liquid chromatography, mass spectrometry, α-glucosidase inhibition

## Abstract

Functional beverages constitute the rapidly increasing part of the functional food section and represent an area with a wide range of products including herbal-based beverages. We carried out screening investigations of the extracts of 85 Rosaceous tea plants. Among the extracts analyzed *Agrimonia asiatica* herb extract demonstrated the highest inhibitory activity against the enzyme α-glucosidase (20.29 µg/mL). As a result of chromato-mass-spectrometric profiling of *A. asiatica* herb with high-performance liquid chromatography with photodiode array and electrospray triple quadrupole mass-spectrometric detection (HPLC-PDA-ESI-tQ-MS) 60 compounds were identified, including catechins, ellagitannins, flavones, flavonols, gallotannins, hydroxycinnamates, procyanidins, most for the very first time. The analysis of the seasonal variation of metabolites in *A. asiatica* herb demonstrated that the phenolic content was highest in summer samples and lower in spring and autumn. HPLC activity-based profiling was utilized to identify compounds of *A. asiatica* herb with the maximal α-glucosidase inhibitory activity. The most pronounced inhibition of α-glucosidase was observed for agrimoniin, while less significant results of inhibition were revealed for ellagic acid and isoquercitrin. The evaluation of phenolic content in *A. asiatica* herbal teas with the subsequent determination of α-glucosidase inhibiting potential was discovered. Maximum inhibition of α-glucosidase was observed for hot infusion (75.33 µg/mL) and the minimum for 30 min decoction (159.14 µg/mL). Our study demonstrated that *A. asiatica* herbal tea is a prospective functional beverage in which dietary intake may help to reduce blood glucose.

## 1. Introduction

Over the past decade, there has been increasing interest in the manufacturing and consumption of functional foods because they ensure considerable health benefits, such as declining the risk of chronic diseases and enhancement of physiological conditions in the human body. In view of the era of pandemics, consumers are interested in functional foods that strengthen the immune system. A recent survey related to the coronavirus disease (COVID) pandemic found that one in five consumers cited immune system support as the top reason for the acquisition of functional foods [1]. However, the accessibility of bioactive ingredients in functional foods may become a problem, as the demand for these products can rise dramatically. Thus, the search for new functional products supporting human health is essential for modern consumers.

Various emerging technologies used in the modern food industry to produce new functional products with health-promoting effects as radio-frequency drying, electro-osmotic dewatering, ultrafiltration, etc. [2,3]. The functional beverage industries present the biggest and rapidly increasing part of the functional food sector comprising of food, beverages, and supplement segments [4]. There are various uses of functional beverages like milk-based drinks, probiotic beverages, energy drinks, caffeinated beverages, sports drinks, herbal-based functional beverages [5]. Herbal beverages (well-known as teas) have gained popularity among consumers who are concerned about their health. They have entered the developing niche market alongside other popular beverages such as tea or coffee which are also made using plant-based materials [6]. The interest of functional beverages has led to the introduction of a number of new herbal teas on the market intended to address particular health problems. For example, a few various types of teas have been reported to reduce blood glucose levels and have antidiabetic effects [7,8,9,10].

Diabetes mellitus (DM) is the most abundant chronic and metabolic illness characterized by an elevation in blood glucose levels because of the absolute or relative insulin deficit. The chronic complications of diabetes are broadly divided into microvascular (neuropathy, nephropathy, and retinopathy) and macrovascular (cardiovascular disease, stroke, peripheral artery disease) [11]. In accordance with the International Diabetes Federation, 327 million people at present suffer from DM, and this figure will reach 438 million by 2045. Type 2 diabetes is the most abundant form of this disturbance, accounting for 90% of the total affected population [12]. Diabetes can be managed using a variety of strategies. Correct treatment demands control in preprandial and postprandial hyperglycemia.

The enzyme α-glucosidase catalyzes the hydrolysis of the α-glycosidic bond of oligosaccharides to release the monosaccharide units from food sources [13,14]. The inhibition of α-glucosidase can delay the release of glucose of oligosaccharides and disaccharides from dietary complex carbohydrates and cause a decrease in postprandial hyperglycemia [15]. Acarbose and other hypoglycemic synthetic α-glucosidase inhibitors have been endorsed for clinical use in the management of DM type 2. Nevertheless, the use of similar medicinal agents is accompanied by undesirable gastrointestinal side-effects (flatulence, stomach ache, diarrhea) and may lead to a limitation in the duration of therapy [16]. So, novel treatment strategies are needed for the prevention or treatment of diabetes mellitus and recently herbal medicines have received the attention of researchers in this area. There is growing evidence that polyphenolic substances exhibit strong α-glucosidase inhibitory activity and are responsible for the reduction of blood glucose levels [17,18]. Among plant polyphenols with antidiabetic properties, ellagitannins have attracted a high degree of interest. These compounds involve structurally complex plant phenolics that are widespread in higher plants, especially in the Rosaceae family, and can reach high molecular masses [19,20].

Although herbal products are generally considered safe due to their natural origin, they are a complex mixture of different substances for which detailed chemical profiling demands serious research [21]. As a part of our continued investigation of antidiabetic plant constituents of Rosaceous plants [22,23,24,25], the present work aimed to perform chromato-mass-spectrometric profiling of *A. asiatica* herb with high-performance liquid chromatography with photodiode array and electrospray triple quadrupole mass-spectrometric detection (HPLC-PDA-ESI-tQ-MS), as well as analysis of *A. asiatica* herb for inhibitory activity against digestive enzyme α-glucosidase. This multisectoral approach is an important basis for promoting the bioactive compounds of *A. asiatica* herb for use in future antidiabetic drugs.

## 2. Materials and Methods

### 2.1. Chemicals

The following chemicals were acquired from Aktin Chemicals Inc. (Chengdu, China): kaempferol-3-*O*-(6″-p-coumaroyl)-glucoside (**56**, tiliroside); Chemwill Asia Co., Ltd. (Beijing, China): agrimoniin (**36**); ChemFaces (Wuhan, China): apigenin 7-*O*-(6″-*O*-malonylglucoside) (**59**); Extrasynthese (Lyon, France): 5-*O*-caffeoyl quinic acid (**14**, neochlorogenic acid); kaempferol-3-*O*-glucoside (**49**); kaempferol-3-*O*-rhamnoside (**54**); luteolin-7-*O*-glucoside (**43**, cynaroside); procyanidin B3 (**12**); quercetin-3-*O*-glucoside (**41**, isoquercitrin); quercetin-3-*O*-rhamnoside (**48**); Sigma-Aldrich (St. Louis, MO, USA): acarbose; apigenin-7-*O*-glucoside (**50**, cosmosiin); apigenin-7-*O*-glucuronide (**51**); bovine serum albumin (BSA); 4-*O*-caffeoylquinic acid (**8**); catechin (**13**); 1,3-di-*O*-caffeoylquinic acid (**26**); 3,5-di-*O*-caffeoylquinic acid (**46**); ellagic acid (**38**); epicatechin (**23**); epicatechin gallate (**37**); epigallocatechin (**15**); 1-*O*-galloyl-glucose (**2**); kaempferol-3-*O*-(6″-*O*-rhamnosyl)-glucoside (**47**, nicotiflorin); luteolin-7-*O*-glucuronide (**44**); procyanidin B1 (**10**); procyanidin B2 (**16**); quercetin-3-*O*-glucuronide (**42**); quercetin-3-*O*-(6″-malonyl)-glucoside (**45**); di-sodium hydrogen phosphate; α-glucosidase from *Saccharomyces cerevisiae* (type 1, 10 U/mg); lithium perchlorate; 4-nitrophenyl-α-d-glucopyranoside; perchloric acid; potassium dihydrogen phosphate; sodium carbonate. 2-Pyrone-4,6-dicarboxylic acid (**6**), pedunculagin (**7**), and potentillin (**32**) were isolated previously from *C. palustre* [22]; tellimagrandins (**11**, **19**, **31**, **40**) were isolated earlier from *Filipendula ulmaria* [23]; gemin A (**34**), agrimonic acid A (**33**), agrimonic acid B (**35**) were isolated from *Potentilla anserina* [24], 6-hydroxyluteolin-7-*O*-glucoside (**28**) was isolated from *Rhaponticum uniflorum* [26].

### 2.2. Plant Material

Plant samples were collected in various Siberian regions (Table S1) and *A. asiatica* was collected in 2018 in the Zabaykalsky Krai, Aga (Agin-Buryat Okrug, 51°12′4.55″ N, 115°9′56.86″ E, 685 m). For a more accurate study of the seasonal fluctuations of phenolic compounds in herb, the samples from three different stages of development were collected in May, July, and September. Individual sampling dates were May 15, July 16, and September 22. All samples were collected between 9 and 11 a.m. The herb samples were sealed in polyethylene bags in the field and placed into a cooler with ice. Upon return to the laboratory, they were air-dried for 10 days at room temperature in a ventilated fume cupboard to a moisture content of 9–12%. Herb samples were stored at 4 °C in the Plant Repository (Institute of General and Experimental Biology). Then 3 obtained samples of the herb of each month were pooled together, resulting in 3 total samples of the seasonal collection. Voucher specimens of *A. asiatica* herb were No. ARo/ag-0911-31/0519 (May samples), ARo/ag-0911-32/0719 (July samples), ARo/ag-0911-32/0919 (September samples). Then the samples were ground in an A11 basic analytical mill (IKA^®^-WerkeGmbh & Co. KG, Staufen, Germany). Subsequently, the samples were sieved up to an average particle diameter of 0.5 mm applying sieving machine ERL-M1 (Zernotekhnika, Moscow, Russia).

### 2.3. Plant Extracts Preparation

To prepare the total extracts of 85 Rosaceous tea plants (powdered samples of herbs (100 g) of *Agrimonia asiatica* Juz., *A. pilosa* Ledeb., *Alchemilla anisopoda* Juz., *A. flavescens* Buser, *A. subcrenata* Buser, *Armeniaca sibirica* (L.) Lam., *Chamaerhodos erecta* (L.) Bunge, *C. grandiflora* (Pall. ex Schult.) Bunge, *Cotoneaster lucidus* Schltdl., *Cotoneaster melanocarpus* Fisch. ex Blytt, *C. mongolicus* Pojark., *C. neopopovii* Czerep., *C. tjuliniae* Pojark. ex Peschkova, *C. uniflorus* Bunge, *Crataegus dahurica* Koehne ex C.K.Schneid., *C. maximowiczii* C.K.Schneid., *C. sanguinea* Pall., *Dasiphora fruticosa* (L.) Rydb., *Dryas grandis* Juz., *D. incisa* Juz., *D. integrifolia* subsp. *crenulata* (Juz.) Kozhevn., *D. oxyodonta* Juz., *D. sumneviczii* Serg., *Dasiphora parvifolia* (Fisch.) Juz., *Fragaria vesca* L., *F. orientalis* Losinsk., *Geum aleppicum* Jacq., *G. rivale* L., *Malus baccata* (L.) Borkh., *Padus avium* Mill., *Potentilla acaulis* L., *P. acervata* Soják, *P. adenotricha* Vodop., *P. anserina* L., *P. arenosa* (Turcz.) Juz., *P. argentea* L., *P. asperrima* Turcz., *P. biflora* Willd. ex Schltdl., *P. chrysantha* Trevir., *P. conferta* Bunge, *P. crantzii* (Crantz) Beck ex Fritsch, *P. desertorum* Bunge, *P. elegans* Cham. & Schltdl., *P. evestita* Th.Wolf, *P. flagellaris* Willd. ex Schltdl., *P. fragarioides* L., *P. kryloviana* Th.Wolf, *P. leucophylla* Pall., *P. longifolia* Willd. ex Schltdl., *P. multifida* L., *P. mujensis* Kurbatski, *P. nivea* L., *P. norvegica* L., *P. nudicaulis* Willd. ex Schltdl., *P. ozjorensis* Peschkova, *P. sanguisorba* Willd. ex Schltdl., *P. saposhnikovii* Kurbatski, *P. sericea* L., *P. stipularis* L., *P. tanacetifolia* Willd. ex Schltdl., *P. tergemina* Soják, *P. verticillaris* Stephan ex Willd., *Prunus pedunculata* (Pall.) Maxim., *Rosa acicularis* Lindl., *R. davurica* Pall., *Rubus arcticus* L., *R. chamaemorus* L., *R. humulifolius* C.A. Mey., *R. matsumuranus* H. Lev. & Vaniot, *R. saxatilis* L., *Sanguisorba alpina* Bunge, *S. officinalis* L., *Sibbaldia adpressa* Bunge, *S. procumbens* L., *Sibbaldianthe bifurca* (L.) Kurtto and T.Erikss., *S. bifurca* subsp. *orientalis* (Juz.) Kurtto & T.Erikss., *Sorbaria pallasii* (G. Don fil.) Pojark., *S. sorbifolia* (L.) A. Braun, *Sorbus sibirica* Hedl., *Spiraea alpina* Pall., *S. aquilegifolia* Pall., *S. dahurica* (Rupr.) Maxim., *S. flexuosa* Fisch. ex Cambess., *S. media* Schmidt, *S. salicifolia* L.) were extracted three times in glass conical flasks (2 L) with water (1 L) with stirring. The extracts have been sonicated for 60 min (50 °C, 100 W, 35 kHz). The extracts obtained were filtrated through a cellulose filter. Consequently, the extracts obtained were combined and then evaporated *in vacuo* until dryness. The obtained extracts were kept at 4 °C until further chemical investigations and analysis of biological activity.

### 2.4. Identification of Phenolic Compounds by High-Performance Liquid Chromatography with Photodiode Array Detection and Electrospray Ionization Triple Quadrupole Mass Spectrometric Detection (HPLC-PDA-ESI-tQ-MS)

The phenolic compounds profiling was realized by reversed-phase high-performance liquid chromatography with photodiode array detection and electrospray ionization mass spectrometry (HPLC-PDA-ESI-tQ-MS) assay using liquid chromatograph LC-20 Prominence coupled with photodiode array detector SPD-M30A (wavelength range 200–600 nm), triple-quadrupole mass spectrometer LCMS 8050 (all Shimadzu, Columbia, MD, USA) and Mastro C18 column (150 × 2.1 mm, 3 μm; Shimadzu GLC, Kyoto, Japan) at the column temperature 30 °C. The following eluents were used: A (0.4% formic acid in water) and B (0.4% formic acid in acetonitrile). The injection volume was 1 μL, and elution flow rate was 80 μL/min. Gradient program: 0.0–2.0 min 5.0–7.5% B, 2.0–7.0 min 7.5–15.0% B, 7.0–11.0 min 15.0–38.0% B, 11.0–14.0 min 38.0–42.0% B, 14.0–20.0 min 42.0–80.0% B, 20.0–25.0 min 80.0–100.0% B, 25.0–35.0 min 100.0–5.0% B. Mass spectrometric detection was used in negative ESI mode (–3 kV source voltage, range of *m*/*z* 100–1900, collision energy 5–40 eV). There were following temperature levels of ESI interface (300 °C), desolvation line (250 °C), and heat block (400 °C). There were following flow rates of nebulizing gas (N_2_, 3 L/min), heating gas (air, 10 L/min), collision-induced dissociation gas (Ar, 0.3 mL/min). The raw data were MS/MS acquired and processed with LabSolutions workstation software (Shimadzu) with the internal LC-MS library. The MS/MS detection parameters of the 44 compounds are shown in Table S2. The identification of compounds was done by the analysis of their retention time, ultraviolet, and mass-spectrometric data (MS, MS/MS) comparing the same parameters with the reference samples and/or literature data.

### 2.5. HPLC-MS Quantification

Quantification of compounds **1–60** were carried out using HPLC-MS data (full scan MS, peak area). The quantifications were realized under the conditions specified in Section 2.4. For the preparation of the stock solutions, 44 reference compounds (**2**, **6**–**16**, **19**, **23**, **26**, **28**, **31**–**51**, **53**–**56**, **58**–**60**) were carefully weighed (10 mg), then separately dissolved in methanol in a W flask (10 mL). An external standard calibration curve was constructed with the use of six data points (100, 50, 25, 10, 5, and 1 µg/mL). The calibration curves were constructed by plotting the MS peak area vs. concentration levels. Correlation coefficients, *r^2^*; standard deviation, *S*_yx_; limits of detection, LOD; limits of quantification, LOQ; linear ranges were computed applying early reference [27]. All analyses were performed in triplicate, data obtained were expressed as mean value ± standard deviation (SD). To prepare a sample solution, 40 mg of the crushed plant was placed in an Eppendorf tube, subsequently, 1 mL of 60% methanol was added. Thereafter the sample was subjected to ultrasonic extraction for 30 min at 50 °C. The weight of the tube was reduced to an initial sign after cooling. For analysis, the extract obtained was filtered through 0.22 μm polytetrafluoroethylene (PTFE) syringe filter and then injected into the HPLC system.

### 2.6. α-Glucosidase Inhibitory Activity

#### 2.6.1. Plant Extracts Analysis

The α-glucosidase inhibiting potential of plant extracts (Section 2.3) was carried out using spectrophotometric assay [28]. Enzyme α-glucosidase was dissolved in phosphate buffer (PBS, pH 6.8) containing 0.2% BSA up to 0.5 U/mL concentration. Sample solution (10 μL) in PBS at various concentrations up 10 to 1000 μg/mL was preblended with 490 μL of PBS and 250 μL *p*-nitrophenyl α-d-glucopyranoside (5 mM) with subsequent 5 min preincubation (37 °C). Then 250 μL α-glucosidase (0.4 U/mL) was added and incubated for 15 min (37 °C). 2000 μL Na_2_CO_3_ (200 mM) was added. The absorbance was measured at 400 nm and calculated as described earlier [28]. Acarbose was applied as a positive control (the half maximal inhibitory concentration IC_50_ 1214.25 ± 24.48 µg/mL) while water was a negative control.

#### 2.6.2. HPLC Based Bioactivity Guided Profiling

Aliquots (100 µL) of *A. asiatica* extract solution (10 mg/mL) were separated under analytical HPLC conditions as described in Section 2.4. The collection of eluates (40 µL) performed every 30 s in 96-well plates. After drying and redissolving the eluates in 10 µL of PBS the eluates were investigated as described earlier [29]. α-Glucosidase was dissolved in PBS which contained BSA (0.2%) up to 0.5 U/mL concentration and then added 125 µL of PBS and 60 µL p-nitrophenyl-α-D-glucopyranoside (5 mM). The samples were incubated at 37 °C for 5 min and then 60 µL α-glucosidase (0.4 U/mL) was added. The samples were incubated at 37 °C for 15 min. Then 50 µL sodium carbonate (200 mM) was added. Absorbance (A400) was determined at 400 nm. The rates (1-A400) were carried out to create HPLC-based bioactivity profiles of *A. asiatica* extract.

### 2.7. Preparation of A. asiatica Herb Infusions and Decoctions

To prepare the infusion of *A. asiatica*, accurately weighed herb (1 g) was mixed with water (20, 50, 80, or 100 °C) in a conical flask and agitated (40 min), followed by the cooling at 20 °C (if required), filtering throw a PTFE filter (0.45 µm) into a volumetric flask (100 mL) and reducing the final volume with distilled water. The decoctions of *A. asiatica* were produced from accurately weighed herb (1 g) after mixing with distilled water (100 mL) in a conical flask, heating on a hotplate, and boiling (15 or 30 min). The resultant decoctions were passed throw a PTFE filter (0.45 µm) into a volumetric flask (100 mL) and the volume was filled with distilled water.

### 2.8. Statistical Analysis

Statistical analyses were carried out with the usage of ANOVA (one-way analysis of variance). The significance of the mean difference was established by Duncan’s multiple range test. Differences were regarded as statistically considerable at *p* < 0.05. The results are presented as mean values ± standard deviations (SD) for three to five replicates.

## 3. Results and Discussion

### 3.1. α-Glucosidase Inhibiting Activity and Ellagitannins Content in 85 Rosaceous Tea Plants

At the preliminary stage of investigation, we decided to screen the most common tea species of the Rosaceae family growing in Siberia to reveal the most active species with the highest α-glucosidase inhibiting activity. The extracts of 85 Rosaceous tea plants were obtained and the content of ellagitannins was determined (Table 1). The maximal ellagitannin content was observed in *Agrimonia asiatica* extract (63.61 mg/g). The high content of ellagitannins (more than 40 mg/g) has also been demonstrated for *Geum aleppicum*, *Sibbaldianthe bifurca*, *Rosa* *acicularis*, *Rubus humilifolius*, *Chamaerhodos grandiflora* species. Further, the study of α-glucosidase inhibitory activity of the extracts investigated was carried out. We decided to divide the species investigated according to their inhibitory activity on α-glucosidase into three groups: active (IC_50_ < 50 μg/mL), medium (IC_50_ 50–100 μg/mL), and low active/inactive (IC_50_ > 100 μg/mL). Based on this criterion, extracts with maximal content of ellagitannins significantly inhibited α-glucosidase and were included in the active group.

**Figure 1:**
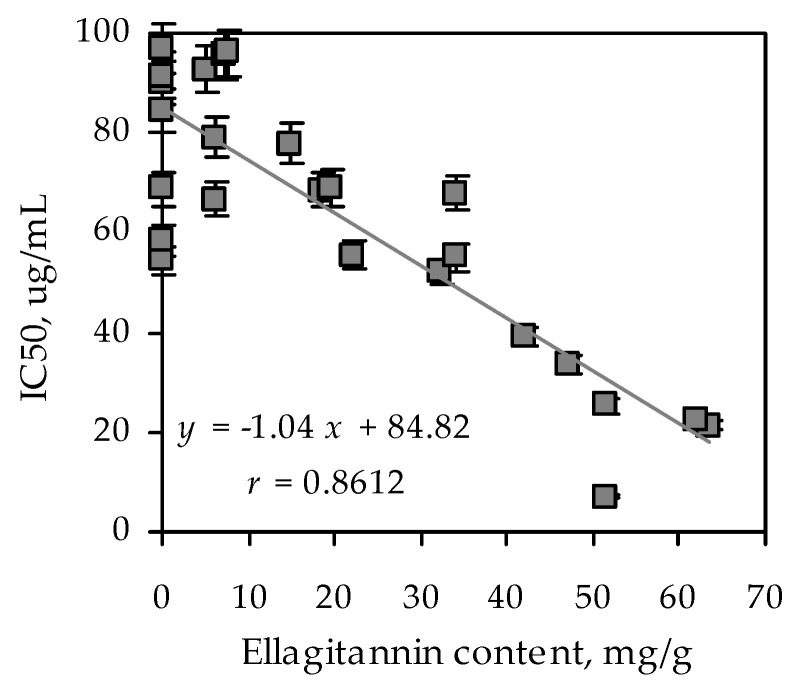
Correlation graph between ellagitannin content (mg/g; independent variable *x* in regression equation) in extracts of Rosaceous species and their α-glucosidase inhibiting activity (IC_50_, μg/mL; dependent variable *y* in regression equation). *r*—correlation coefficient.

As a result of enzymatic analysis, it was revealed that *A. asiatica* extract inhibited α-glucosidase with the highest IC_50_ value (20.29 µg/mL). Acarbose inhibited α-glucosidase with IC_50_ value 1214.25 µg/mL. To reveal the links among the ellagitannins content and biological activity, linear regression analysis was applied (Figure 1). A strong and positive correlation was observed between ellagitannins content and enzyme inhibiting activity (r = 0.8612). The findings indicated that ellagitannins contributed to the inhibition of the enzyme α-glucosidase. Based on the data obtained we decided to choose *A. asiatica* as the herb for further investigation. In addition, there is information about the antidiabetic activity of the genus *Agrimonia* in the literature [30,31]. The chemical composition of this plant species is insufficiently studied. Only the detection of ursolic acid in *A. asiatica* herb has been studied to this point [32]. To characterize the chemical composition of the genus *Agrimonia*, we compiled a review on the content of chemical compounds in representatives of this genus. According to the literature data, the known information about the chemical composition of *Agrimonia* species relates to *A. pilosa* and *A. eupatoria* in general. Thus, the presence of phenolic acids, resorcinols, phloroglucinols, hydroxycinnamates, coumarins, chromones, flavones, flavonols, flavanonols, catechins, proanthocyanidins, ellagitannins, sterols, triterpenoids, etc. were established (Table 2).

Flavonols, flavones, and flavanonols were the most studied phenolic compounds of the *Agrimonia* genus. Some chemical information on *A. procera* (hydroxycynnamates, flavones, flavonols, and ellagitannins) was provided.

### 3.2. Ellagitannins and Other Phenolics of Agrimonia asiatica: LC-MS Seasonal Profile

**Figure 2:**
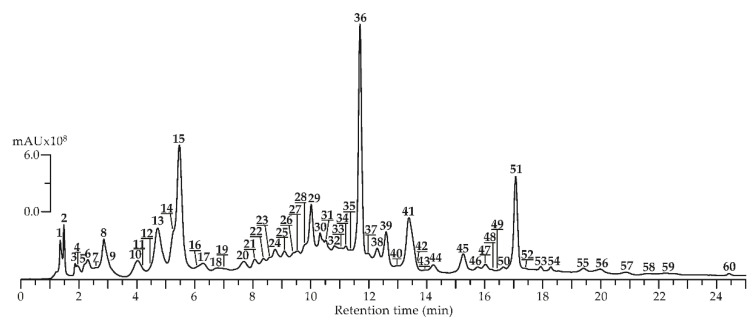
High-Performance Liquid Chromatography with Photodiode Array Detection and Electrospray Ionization Triple Quadrupole Mass Spectrometric Detection (HPLC-PDA-ESI-tQ-MS) chromatogram (Total Ion Chromatogram or TIC mode, negative ionization) of *A. asiatica* herbal extract. Compounds are numbered as listed in Table 3.

To identify potential tendencies in the phenolic constituent profile, *Agrimonia asiatica* herb samples were collected in May, July, and September, and methanol extracts were obtained. These extracts were analyzed by reversed-phase high-performance liquid chromatography with photodiode array detection and electrospray ionization triple quadrupole mass spectrometric detection (HPLC-PDA-ESI-tQ-MS) in negative ionization mode. HPLC-MS chromatogram is shown in Figure 2 and chromatographic, mass-spectrometric data (ESI-MS), and seasonal presence/content of compounds found in *A. asiatica* herb are in Table 3.

By comparison the retention times (t_R_), ultraviolet (UV) and ESI-MS spectra with those of reference substances and literature data, 60 compounds were discovered in all plant samples, including four catechins (**13**,**15**,**23**,**37**), 18 ellagitannins (**7**,**9**,**11**,**18**,**19**,**21**,**22**,**24**,**25**,**27**,**29**,**31**–**36**,**40**), 20 flavones/flavonols (**28**,**39**,**41**–**45**,**47**–**56**,**58**–**60**), four gallotannins (**2**–**5**), four hydroxycinnamates (**8**,**14**,**26**,**46**), five procyanidins (**10**,**12**,**16**,**20**,**30**), one sugar (**1**) and four various phenolics (**6**,**17**,**38**,**57**).

Four chromatographic peaks with a similar UV profile typical of caffeoyl derivatives (caffeoylquinic acids) were detected in *A. asiatica*. The charge on deprotonated ions made it possible to identify them as mono-*O*-caffeoylquinic acids with *m*/*z* 353 (**8**,**14**) and di-*O*-caffeoylquinic acids with *m*/*z* 515 (**26**,**46**). Compared with reference standards 4-*O*-caffeoylquinic (**8**), 5-*O*-caffeoylquinic (**14**), 1,3-di-*O*-caffeoylquinic (**26**), 3,5-di-*O*-caffeoylquinic acids (**46**) were found. Mono-*O*-caffeoylquinic acids (**8**,**14**) were detected in *A. eupatoria* and *A. procera* previously [36,45], while di-*O*-caffeoylquinic acids (**26**,**46**) were discovered in the genus *Agrimonia* for the first time. It should be noted that all hydroxycinnamates were present in all seasonal samples of *A. asiatica*. The content of 4-*O*-caffeoylquinic acid (**8**) reached a maximum in the July sample (18.59 mg/g) and slightly decreased in the September sample (16.37 mg/g). However, the content of 3,5-di-*O*-caffeoylquinic acid (**46**) significantly decreased more than 8 times in September samples (0.31 mg/g) compared with July samples (2.59 mg/g).

Twenty flavone/flavonol glycosides were identified as derivatives of quercetin (**39**,**41**,**42**,**45**,**48**,**55**), kaempferol (**47**,**49**,**52**,**54**,**56**), apigenin (**50**,**51**,**59**,**60**) and luteolin (**28**,**43**,**44**,**53**,**58**). Flavonol glycosides were represented as derivatives of quercetin and kaempferol [36,56].

Quercetin-3-*O*-(6″-*O*-rhamnosyl)-glucoside (**39**), quercetin-3-*O*-glucoside (isoquercitrin, **41**), quercetin-3-*O*-glucuronide (miquelianin, **42**), quercetin-3-*O*-(6″-*O*-malonyl)-glucoside (**45**), quercetin-3-*O*-rhamnoside (quercitrin, **48**), quercetin-3-*O*-(6″-*O*-*p*-coumaroyl)-glucoside (**55**) standards made it possible to unambiguously identify these six flavonol glycosides in *A. asiatica* extract. Isoquercitrin (**41**) and quercitrin (**48**) have already been characterized as components of *A. eupatoria*, *A. procera* and *A. pilosa* [36,58]. Whereas quercetin-3-*O*-(6″-*O*-rhamnosyl)-glucoside (**39**), quercetin-3-*O*-glucuronide (miquelianin, **42**), quercetin-3-*O*-(6″-*O*-malonyl)-glucoside (**45**), quercetin-3-*O*-(6″-*O*-*p*-coumaroyl)-glucoside (**55**) were discovered in genus *Agrimonia* for the first time. Four kaempferol derivatives were detected as kaempferol-3-*O*-(6″-*O*-rhamnosyl)-glucoside (**47**), kaempferol-3-*O*-glucoside (astragalin, **49**), kaempferol-3-*O*-rhamnoside (afzelin, **54**), kaempferol-3-*O*-(6″-*O*-*p*-coumaroyl)-glucoside (tiliroside, **56**) by comparison with standards. Compound **52** was identified as kaempferol-*O*-malonyl-*O*-hexoside in accordance with UV and ESI-MS spectra and literature data. The negative mass spectrum displayed a deprotonated ion [M-H]^-^ with *m*/*z* 533, as well as demalonated fragment with *m*/*z* 447, and the aglycone fragment with *m*/*z* 285 caused the removal of a hexose particle [69]. Astragalin (**49**) was revealed earlier in *A. eupatoria* [55], *A. pilosa* [56], *A. procera* [36]. Afzelin (**54**) was detected in *A. eupatoria* [55] and *A. pilosa* [56]. Tiliroside (**56**) was previously identified in *A. eupatoria* [34] and *A. pilosa* [57]. Kaempferol-3-*O*-(6″-*O*-rhamnosyl)-glucoside (**47**) was discovered in *A. asiatica* for the first time in this work. Five luteolin derivatives were characterized as 6-hydroxyluteolin-7-*O*-glucoside (**28**), luteolin-7-*O*-glucoside (cynaroside, **43**), luteolin-7-*O*-glucuronide (**44**), luteolin-3-*O*-(6″-*O*-malonyl)-glucoside (**53**), luteolin-7-*O*-(6″-*O*-*p*-coumaroyl)-glucoside (**58**) after comparison of t_R_, UV, and mass-spectrometric data (MS, MS/MS) with the reference standards. Four apigenin derivatives: apigenin-7-*O*-glucoside (cosmosiin, **50**), apigenin-7-*O*-glucuronide (**51**), apigenin-7-*O*-(6″-*O*-*p*-malonyl)-glucoside (**59**) and apigenin-7-*O*-(6″-*O*-p-coumaroyl)-glucoside (**60**) were detected after comparison with standards. Two luteolin derivatives cynaroside (**43**) and luteolin-7-*O*-glucuronide (**44**) and two apigenin derivatives cosmosiin (**50**) and apigenin-7-*O*-glucuronide (**51**) have already been characterized as components of *A. eupatoria*, *A. pilosa* and *A. procera* [36,45,51,52,53]. 6-hydroxyluteolin-7-*O*-glucoside (**28**), luteolin-3-*O*-(6″-*O*-malonyl)-glucoside (**53**), luteolin-7-*O*-(6″-*O*-*p*-coumaroyl)-glucoside (**58**), apigenin-7-*O*-(6″-*O*-*p*-malonyl)-glucoside (**59**) and apigenin-7-*O*-(6″-*O*-*p*-coumaroyl)-glucoside (**60**) were discovered in the *Agrimonia* genus for the first time. According to the data obtained, the presence of flavone/flavonol glycosides is observed in all seasonal samples of *A. asiatica*. However, there were curious differences between the different derivatives of flavones and flavonols. It was noticed that the presence of some flavones, in particular apigenin derivatives, was detected in all seasonal samples of the *A. asiatica* and even an increase in the content of some compounds (apigenin-7-*O*-glucoside, **50**; apigenin-7-*O*-glucuronide, **51**) was observed in the September samples. Other representatives of flavones, luteolin derivatives, were not present in all seasonal samples of the *A. asiatica*. It is difficult to trace the accumulation trend for quercetin derivatives, since the peak accumulation of some compounds occurs in May samples (quercetin-3-*O*-(6″-*O*-malonyl)-glucoside, **45**), July samples (quercetin-3-*O*-glucoside, **41**) and even September samples (quercetin-3-*O*-(6″-*O*-rhamnosyl)-glucoside, **39**). Other representatives of flavonols, kaempferol derivatives were present in all seasonal samples, and the maximum content of some compounds is observed in September samples (kaempferol-3-*O*-(6″-*O*-rhamnosyl)-glucoside, **47**; kaempferol-3-*O*-rhamnoside, **54**).

Hydrolyzable tannins of *A. asiatica* were presented by both groups—ellagitannins and gallotannins. Ellagitannins are esters of hexahydroxydiphenoyl (HHDP) groups with a sugar core and often contain galloyl groups. Gallotannins consist of a sugar substituted only with galloyl groups [70]. Ellagitannins of various types: *C*-glucosidic (casuariin, **9**), hexahydroxydiphenoyl glucose (pedunculagin, **7**), hexahydroxydiphenoyl-galloyl-glucose (tellimagrandins I_1_ (**11**), I_2_ (**19**), II_1_ (**31**), II_2_ (**40**), potentillin (**32**)), dehydrodigalloyl (agrimoniin (**36**), gemin A (**34**), agrimonic acids A (**33**) and B (**35**)) were found in *A. asiatica* herb by comparison their properties with standards. Compound **18** was identified as tri-*O*-galloyl-*O*-hexahydroxydiphenoyl glucose in accordance with UV, mass-spectrometric data (MS, MS/MS), and literature data. The negative mass spectrum showed a deprotonated ion [M-H]^-^ with *m*/*z* 937 and the fragmentation patterns corresponded to the loss of gallic acid units, HHDP units, and the loss of a glucosyl moiety [71]. Di-*O*-galloyl-*O*-hexahydroxydiphenoyl-glucose (**22**,**25**) was identified based on the relationship between the supposed parent deprotonated ion at *m*/*z* 785 and fragments having *m*/*z* values corresponding to the losses of galloyl units, glucosyl units, and HHDP units [72]. Compounds **21**, **27**, **29** were identified as bis-*O*-hexahydroxydiphenoyl glucose and were possible isomers of pedunculagin (**7**) or casuariin (**9**). These compounds were isomeric and displayed a parent peak at *m*/*z* 783, yielding main fragment ions at *m*/*z* 481 [(M-H)−302]^-^ (loss of HHDP) and 301 [(M-H)−482]^-^ (loss of HHDP-glucose), whose fragmentation pattern corresponds to a bis-HHDP-glucose structure [73,74]. Compound **24** was identified as hexahydroxydiphenic acid in accordance with UV and ESI-MS spectra and literature data. The negative mass spectrum showed a deprotonated ion [M-H]^-^ with *m*/*z* 337 [71]. Pedunculagin (**7**), agrimoniin (**36**), potentillin (**32**), agrimonic acids A (**33**), and B (**35**) were found in *A. pilosa* earlier [63,64]. Agrimoniin (**36**) was also found in *A. eupatoria* [45] and *A. procera* [36]. Thus casuariin (**9**), tellimagrandins I_1_ (**11**), I_2_ (**19**), II_1_ (**31**), II_2_ (**40**), and gemin A (**34**) were described for the genus *Agrimonia* for the first time.

Significant changes in the ellagitannin seasonal profile were observed. It was noticed that the presence of some ellagitannins was detected in all seasonal samples of *A. asiatica*, while other ellagitannins were revealed only in samples of a certain season. Thus, the presence of ellagitannin of hexahydroxydiphenoyl glucose pedunculagin (**7**) and *C*-glucosidic casuariin (**9**) groups was noticed for all seasonal samples of *A. asiatica* (May, July, September), while ellagitannins of hexahydroxydiphenoyl-galloyl-glucose group tellimagrandins I_1_ (**11**), I_2_ (**19**), II_1_ (**31**), II_2_ (**40**) were revealed only in May and July samples of *A. asiatica*. It may be because *C*-glucosidic ellagitannin casuariin (**9**) is supposed to be biosynthesized from pedunculagin (**7**) due to its almost unchanged combination with pedunculagin and/or tellimagrandin I_1_ (**11**), I_2_ (**19**), and is a plausible precursor. Pedunculagin (**7**) can be considered as the product of oxidative binding between two galloyl groups in tellimagrandin I (**11**,**19**). A similar trend was observed for the seasonal fluctuations in the content of these compounds in leaves of *Liquidambar formosana* (Hamamelidaceae) [75]. The synthesis of casuariin (**9**) in the leaves of *L. formosana* in autumn was followed by a decrease and then by the complete disappearance of tellimagrandins I (**11**,**19**) and II (**31**,**40**), which were abundant in the young leaf in spring. This seasonal change is in accordance with the probability theory that this *C*-glucosidic tannin is biosynthesized from the gallotannins and ellagitannins which were found in the young leaves [76].

Ellagitannin agrimoniin (**36**) was the dominant compound of *A. asiatica* herb. Its content was the highest in July samples (114.18 mg/g) during mass flowering and then sharply decreased in September samples by more than 4 times (27.32 mg/g). A similar phenomenon was observed for the genus *Alchemilla*. In *A. vulgaris* and *A. mollis* the content of agrimoniin (**36**) increased by the end of the mass flowering period by more than 100% [77]. The presence of potentillin (**32**) in all seasonal samples is probably because it is a main intermediate metabolite in the production of agrimoniin. Agrimoniin (**36**) is a dimeric ellagitannin in which hydroxyl groups on the two glucose cores are esterified by a dehydrodigalloyl group and four hexahydroxydiphenoyl groups. Agrimoniin (**36**) may have been formed by intermolecular C–O oxidative binding between galloyl groups in two molecules of potentillin (**32**) [64].

Gallotannins were the second group of hydrolysable tannins and were presented in *A. asiatica* herb by 1-*O*-galloyl glucose (**2**) and *O*-galloyl-glucoses (**3**,**4**,**5**). Gallotannins are turned into to ellagitannins through oxidative binding of the galloyl groups. Several ellagitannin derivatives form dimers after intermolecular binding with other hydrolyzable tannin molecules [78]. The content of galloylglucoses (**2**,**3**,**5**) was higher in May samples than in the samples collected in July during the mass flowering period. Galloylglucoses are predecessors to other more complex hydrolyzable tannins. Consimilar season tendency for galloylglucoses has been reported in the investigations of *Geranium sylvaticum* [79], *Quercus robur* [80], and *Betula pubescens* [81]. In accordance with literature data, the structures of tree leaf hydrolyzable tannins vary within the vegetation period. These variations follow the biogenetic pathway. Thus, the simple galloylglucose predecessors revealed in springtime are converted to more complex compounds. These complex structures contain more galloyl and oxidatively binded HHDP groups during the vegetation season. In contrast to woody plants, there is an assumption that hydrolyzable tannins of herbaceous plants generally preserve their chemical structures until leaves destruction [82]. Nevertheless, research results demonstrate that hydrolyzable ellagitannins can be converted during the vegetation season in herbaceous plants, as has been observed in the case herb of *A. asiatica*. The contents of galloylglucoses, that are predecessors to more complex hydrolyzable tannins, were higher in May samples, and the amount of further modified ellagitannins increased towards the flowering period (July sample). So, to maximize the isolated content of ellagitannins, *A. asiatica* herb must be harvested during the flowering period (July).

### 3.3. α-Glucosidase Inhibiting Activity of A. asiatica Extract: HPLC Activity-Based Profiling

In natural product research, the finding of new bioactive components useful for drug discovery is the main goal. This requires the early detection of bioactive substances in complex matrices to isolate only the necessary components [83]. A common procedure involves searching for substances from complex matrices such as extracts, including biological screening followed by activity guided fractionation [84]. Such approaches mainly consist of the post-column collecting of microfractions, drying them for solvent removal, and biological assessment of the microfractions. Ultimately, the activity measured for each microfraction, is compared to the HPLC profile to reveal the LC peaks that are responsible for the activity [83].

**Figure 3:**
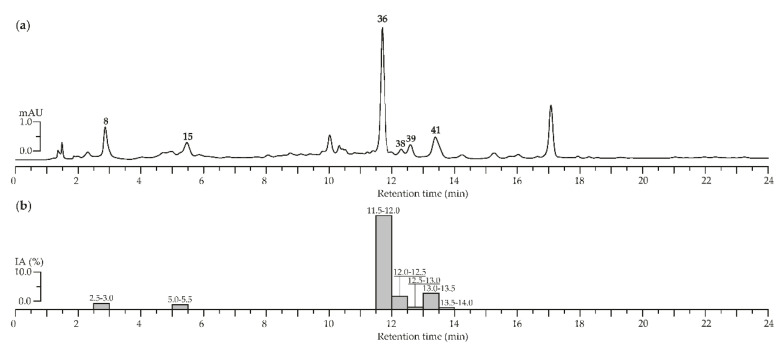
(**a**) High-Performance Liquid Chromatography with Photodiode Array Detection (HPLC-PDA) chromatograms of *A. asiatica* extract at 270 nm. Compounds numbered as **8** (4-*O*-caffeoylquinic acid), **15** (epigallocatechin), **36** (agrimoniin), **38** (ellagic acid), **39** (quercetin-3-*O*-(6″-*O*-rhamnosyl)-glucoside), and **41** (quercetin-3-*O*-glucoside). (**b**) α-Glucosidase inhibiting activity (IA, as a percentage) of HPLC fractions of *A. asiatica* extract after microfractionation technique. Bars are labeled as retention time range (min).

To characterize the chemical profile of *A. asiatica* herb extract microcolumn reversed-phase HPLC-PDA procedure was applied (Figure 3a). We compared the data received with those of reference substances 4-*O*-caffeoylquinic acid (**8**), epigallocatechin (**15**), agrimoniin (**36**), ellagic acid (**38**), quercetin-3-*O*-(6″-*O*-rhamnosyl)-glucoside (**39**), and quercetin-3-*O*-glucoside (isoquercitrin, **41)** were revealed. For identification, the components of interest of *A. asiatica* herb with high α-glucosidase inhibitory activity, the extract was subjected by HPLC activity-based profiling. To detect of anti-α-glucosidase inhibitors in *A. asiatica* herb extract, the procedure of small-scale semi-preparative microfractionation by reversed-phase HPLC was applied. This resulted in 48 fractions of 30 s each that were transferred to a deep-well microtiter plate. Subsequently, microfractions were dried and redissolved in a buffer solution. Thereafter, an enzyme solution was added to estimate the inhibition of α-glucosidase by the eluate. The optical densities at 400 nm (A_400_) as (1-A_400_) values of microfractions after post-column derivatization are displayed in Figure 3b.

The main inhibition was observed in the time window of 11.5 to 13.5 min, corresponding to 5 fractions. The α-glucosidase inhibition activity measured for each microfraction was compared with the HPLC profile to localize the corresponding chromatogram peaks. The most pronounced inhibition of α-glucosidase was observed for agrimoniin (**36**), while less significant inhibition was revealed for ellagic acid (**38**) and quercetin-3-*O*-glucoside (isoquercitrin, **41**). In this way, ellagitannin agrimoniin (**36**) from the extract of *A. asiatica* herb is considered the responsible compound for the inhibition of α-glucosidase previously detected in screening investigations. Earlier we proved high inhibitory activity of agrimoniin (**36**) from *Comarum palustre* herb extract relative to α-glucosidase [29]. A possible mechanism of the inhibitory activity against α-glucosidase of ellagitannins from the extract analyzed could be tannin–protein coupling. There are changes in the conformation of the enzyme and therefore the activity of α-glucosidase decreases due to association and precipitation [85,86].

Thus, the microfractionation of crude plant extracts, particularly *A. asiatica*, makes it possible to directly evaluate the bioactivities of LC-peaks within their biological matrices. By miniaturizing enzymatic assays in a deep-well microtiter plate, HPLC-based biological activity profiling has become accessible for many types of analyzes and is supplementary to metabolite profiling, enabling bioactive components to easily be localized and partly identified. In this way, such methods should accelerate the detection of compounds from plant extracts and help rationalize the classical approach based on biological activity, which was considered as too slow and difficult for the pharmaceutical industries [87].

### 3.4. Stability of A. asiatica Phenolics in Water Media: Comparison of Infusion and Decoction Composition and Their Anti-α-Glucosidase Activity

When natural biologically active components are extracted from plant materials, the main disadvantages are associated with possible decomposition and/or changes in chemical structures. As such, the extraction procedures must be chosen carefully. So, we decided to compare phenolic content in *A. asiatica* herb infusions and decoctions depending on the method of obtaining herbal tea (Table 4). Herbal drinks are beverages made from the infusion or decoction of herbs, spices, fruits, or other plant materials, that are served cold or hot [88]. In decoction, the plant material is boiled, or kept at high temperatures, in water for a certain time. An infusion is obtained by adding water to a certain amount of plant material and leaving the mix to brew, similar to the preparation of tea [89].

The maximal content of ellagitannins (38.20 mg/100 mL) was observed in the infusion obtained by the extraction of herb *A. asiatica* with water at 80 °C. On the contrary, in the decoction obtained by extraction of herb *A. asiatica* for 15 min, a significant decrease in ellagitannins (4.75 mg/100 mL) content was revealed. During extraction for 30 min, complete degradation and trace amounts of ellagitannins were detected. However, for both decoctions, a significant increase in ellagic acid was observed. It has previously been reported earlier that ellagitannins may be unstable when processed at higher temperatures, leading to their degradation to ellagic acid [90]. Our findings confirm early data for *A. eupatoria* herb that the degradation products of agrimoniin are not present in large quantities if the effect of temperature is limited in time [36,45].

The total content of hydroxycinnamates gradually increased in infusions from 3.86 mg/100 mL to 6.65 mg/100 mL as the temperature of the extract increased. Then, when analyzing the decoctions, a slight decrease in the content of hydroxycinnamates was revealed. Li et al. had previously studied the thermal stability of mono- and di-caffeoylquinic acids [91]. It was found that after heating for 1 h, the amount of three mono-caffeoylquinic acids was reduced by about 10% and the amount of di-caffeoylquinic acids was decreased by about 20%. Thus, the proposed extraction method was heating for less than an hour in boiling water, to minimize artifacts in the analyzes of mono- and di-caffeoylquinic acids.

The highest content of flavones and flavonols (33.78 mg/100 mL) was observed in the decoction obtained by boiling the herb of *A. asiatica* for 30 min and minimal content (19.01 mg/100 mL) was observed for cold infusion. It is interesting, that contents of rhamnosyl-glucosides of quercetin and kaempferol, as well as glucosides of quercetin and apigenin, glucuronides of luteolin and apigenin and kaempferol-3-*O*-rhamnoside increased with rising extraction temperature. Meanwhile, the content of quercetin-3-*O*-(6″-*O*-malonyl)-glucoside and luteolin-3-*O*-(6″-*O*-malonyl)-glucoside decreased to trace amounts in samples obtained by boiling infusion and decoctions. This simple phenomenon was described by Katsube et al. where they proved that heat treatment at high temperatures induced the release of CO_2_ from the malonyl group of quercetin-3-*O*-(6″-*O*-malonyl)-glucoside, resulting in the formation of quercetin-3-*O*-(6-*O*-acetyl)-glucoside [92]. However, quercetin-3-*O*-(6-*O*-acetyl)-glucoside was not found in the *Agrimonia* tea infusions and decoctions in the present study.

The α-Glucosidase inhibiting potential of infusions and decoctions obtained was studied. As shown previously, the correlation analysis showed a univocal relationship between the concentration of ellagitannins in the plant extract and its inhibitory activity. So, the *A. asiatica* herb was characterized by a high content of agrimoniin, and therefore high inhibitory activity against digestive enzyme α-glucosidase in dosage forms with the highest expected content of this component. The maximum inhibition of α-glucosidase was observed for hot infusion (75.33 µg/mL), and the minimum for 30 min decoction (159.14 µg/mL). Probably due to the high content of agrimoniin in the hot infusion, this dosage form showed the greatest activity in inhibiting α-glucosidase. Thus, the quantitative and qualitative changes of the phenolic profile in *Agrimonia* tea infusions and decoctions are strongly influenced by heating temperatures. A similar analysis of functional polyphenols in the herb of *A. asiatica* from this research is useful for standardization and physiological estimation of a perspective functional beverage—*Agrimonia* herbal tea. To meet the legitimate demands (recommended daily allowance, reference nutritional intake), in vitro investigations should be maintained by in vivo researches to define the bioavailability and bioaccessibility of *Agrimonia* herbal tea. There is currently a renaissance in food technology that allows the creation of new functional products with health-promoting effects from Conilon and Arabica coffee flowers [93], green tea [94,95,96,97,98,99,100], olive [101], Sabah snake grass [102], etc. It is suggested that *Agrimonia* herbal tea may be part of the regular diet of people with diabetes to improve health or decrease the risk of this illness that goes beyond basic nutritional functions.

## 4. Conclusions

In this paper, a screening investigation of the extracts of 85 Rosaceous tea plants was carried out and *Agrimonia asiatica* was found to be the extract with the highest inhibitory activity against the α-glucosidase enzyme. As a result of chromato-mass-spectrometric profiling of *A. asiatica* herb with high-performance liquid chromatography with photodiode array and electrospray triple quadrupole mass-spectrometric detection, 60 compounds were identified, including catechins, ellagitannins, flavones, flavonols, gallotannins, hydroxycinnamates, procyanidins. To identify the compounds of *A. asiatica* herb with the highest α-glucosidase inhibitory activity *A. asiatica* herb extract was subjected to HPLC activity-based profiling which resulted in 5 active fractions. The most pronounced inhibition of α-glucosidase was observed for agrimoniin, while less significant results of inhibition were revealed for ellagic acid and isoquercitrin. The comparison of phenolic content in *A. asiatica* herb infusions and decoctions depending on the method of obtaining herbal tea with the subsequent determination of α-glucosidase inhibiting potential was also carried out. It was found that maximum inhibition of α-glucosidase was observed for hot infusion and the minimum for 30 min decoction. This investigation demonstrated that *Agrimonia* herbal tea is a feasible functional beverage and its dietary intake may help to decrease postprandial hyperglycemia.

## Figures and Tables

**Table 1:** α-Glucosidase inhibiting activity (IC_50_, μg/mL ± S.D.)* and ellagitannin content (ET, mg/g of dry extract weight ± S.D.) for extracts from 85 Rosaceous tea plants.

Species	IC_50_, μg/mL	ET, mg/g	Group
*Agrimonia asiatica*	20.29 ± 0.42	63.61 ± 1.59	Active (IC_50_ < 50 μg/mL)
*Geum aleppicum*	29.31 ± 0.44	52.03 ± 1.43	
*Sibbaldianthe bifurca*	31.16 ± 0.50	51.45 ± 1.28	
*Rosa* *acicularis*	32.33 ± 0.54	50.04 ± 1.12	
*Rubus humulifolius*	33.63 ± 0.71	47.05 ± 0.99	
*Chamaerhodos grandiflora*	39.26 ± 0.70	41.94 ± 0.83	
*Waldstenia ternata*	52.18 ± 1.04	32.14 ± 0.64	Medium (IC_50_ 50–100 μg/mL)
*Potentilla conferta*	54.37 ± 1.02	tr.	
*Rubus matsumuranus*	55.12 ± 1.12	34.16 ± 0.71	
*Dasiphora fruticosa*	55.45 ± 1.27	22.18 ± 0.51	
*Dryas oxyodonta*	58.48 ± 1.28	tr.	
*Potentilla tergemina*	66.71 ± 1.41	6.30 ± 0.12	
*Agrimonia pilosa*	67.82 ± 1.36	34.27 ± 0.82	
*Dasiphora parvifolia*	68.93 ± 1.57	18.45 ± 0.47	
*Potentilla longifolia*	68.86 ± 1.51	tr.	
*Potentilla stipularis*	68.46 ± 1.51	19.57 ± 0.45	
*Sanguisorba officinalis*	77.88 ± 1.75	14.85 ± 0.31	
*Potentilla fragarioides*	79.03 ± 1.58	6.34 ± 0.11	
*Crataegus sanguinea*	84.36 ± 1.94	tr.	
*Cotoneaster melanocarpus*	90.12 ± 2.16	tr.	
*Potentilla nudicaulis*	91.52 ± 2.21	tr.	
*Sibbaldia procumbens*	92.61 ± 2.31	5.22 ± 0.10	
*Fragaria vesca*	95.47 ± 1.90	7.36 ± 0.14	
*Sanguisorba alpina*	96.07 ± 2.49	7.55 ± 0.11	
*Potentilla tanacetifolia*	96.93 ± 2.52	tr.	
60 Rosaceous species ^b^	>100	tr.	Low active / inactive (IC_50_ > 100 μg/mL)

* Acarbose was used as a reference compound (IC_50_ 1214.25 ± 24.48 μg/mL). ^b^ Listed in Table S1. tr.—traces (<0.01 mg/g).

**Table 2:** Known compounds found in *Agrimonia* species (literature data).

Compound	Species	Found in [ref.]	
		Herb	Roots
Phenolic Acids			
*p*-Hydroxybenzoic acid	*A. pilosa*	[33]	
Protocatechuic acid	*A. eupatoria*	[34]	
	*A. pilosa*	[33]	
Isovanillic acid	*A. pilosa*	[33]	
Gallic acid	*A. eupatoria*	[35]	
	*A. pilosa*	[33]	
Ellagic acid	*A. eupatoria*	[36]	
	*A. pilosa*	[37]	[38]
Ellagic acid-4-*O*-Xyl*p*	*A. pilosa*		[38]
Resorcinols			
Agrimopiloside A	*A. pilosa*	[39]	
Agrimopiloside B	*A. pilosa*	[39]	
1-Butanoyl-3,5-dimethyl-phloroglucinyl-6-*O*-Glc*p Phloroglucinols*	*A. pilosa*	[40]	
Agrimol A	*A. pilosa*		[41]
Agrimol B	*A. pilosa*	[33]	[41]
Agrimol C	*A. pilosa*		[42]
Agrimol D	*A. pilosa*	[33]	[41]
Agrimol F	*A. pilosa*		[42]
Agrimol G	*A. pilosa*		[42]
Agrimophol	*A. pilosa*		[41]
Agripinol A	*A. pilosa*	[43]	
Agripinol B	*A. pilosa*	[43]	
Agripinol C	*A. pilosa*	[43]	
Pseudoaspidin	*A. pilosa*	[43]	[44]
α-Cosin	*A. pilosa*	[43]	
Hydroxycinnamates			
*p*-Coumaric acid	*A. eupatoria*	[34]	
	*A. pilosa*	[33]	
3-*O*-*p*-Coumaroylquinic acid	*A. eupatoria*	[45]	
	*A. procera*	[36]	
1-*O*-Caffeoylquinic acid	*A. eupatoria*	[36]	
	*A. procera*	[36]	
3-*O*-Caffeoylquinic acid	*A. eupatoria*	[36]	
	*A. procera*	[36]	
4-*O*-Caffeoylquinic acid	*A. eupatoria*	[36]	
	*A. procera*	[36]	
5-*O*-Caffeoylquinic acid	*A. eupatoria*	[45]	
	*A. procera*	[36]	
Coumarins			
Agrimonolide	*A. pilosa*	[40]	[46]
Agrimonolide-6-*O*-Glc*p*	*A. pilosa*	[47]	[48]
(3*S*)-Agrimonolide-6-*O*-(6′-*O*-Ara*f*)-Glc*p*	*A. pilosa*	[49]	
Desmethylagrimonolide-6-*O*-Glc*p*	*A. pilosa*	[50]	
(3*S*)-Desmethylagrimonolide-4′-*O*-Glc*p*	*A. pilosa*	[49]	
(3*S*,4*R*)-4-Hydroxyagrimonolide-6-*O*-Glc*p*	*A. pilosa*	[49]	
5,7-Dihydroxy-2-propylchromone-7-*O*-Glc*p Chromones*	*A. pilosa*	[50]	
Takanechromone C	*A. pilosa*	[50]	
Petiolin E	*A. pilosa*	[33]	
Flavones			
Apigenin	*A. eupatoria*	[51]	
Apigenin-7-*O*-Glc*p* (= cosmosiin)	*A. eupatoria*	[51]	
	*A. pilosa*	[52]	
	*A. procera*	[36]	
Apigenin-7-*O*-GlcA*p*	*A. eupatoria*	[45]	
	*A. pilosa*	[53]	
	*A. procera*	[36]	
Apigenin-7-*O*-(6″-Me)-GlcA*p*	*A. pilosa*	[54]	
Apigenin-6-*C*-Glc*p* (= isovitexin)	*A. eupatoria*	[34]	
Apigenin-8-*C*-Glc*p* (= vitexin)	*A. eupatoria*	[45]	
Luteolin	*A. eupatoria*	[51]	
Luteolin-7-*O*-Glc*p* (= cynaroside)	*A. eupatoria*	[51]	
	*A. pilosa*	[52]	
	*A. procera*	[36]	
Luteolin-7-*O*-GlcA*p*	*A. eupatoria*	[45]	
	*A. pilosa*	[53]	
	*A. procera*	[36]	
Luteolin-7-*O*-(6″-Me)-GlcA*p*	*A. pilosa*	[37]	
Luteolin-7-*O*-(6″-But)-GlcA*p*	*A. pilosa*	[37]	
Luteolin-3′-*O*-Glc*p*	*A. pilosa*	[37]	
Pinocembrin	*A. pilosa*	[33]	
Flavonols			
Kaempferol	*A. eupatoria*	[55]	
	*A. pilosa*	[56]	
Kaempferol-3-*O*-Ara*p*	*A. pilosa*	[33]	
Kaempferol-3-*O*-Rha*p* (= afzelin)	*A. eupatoria*	[55]	
	*A. pilosa*	[56]	
Kaempferol-3-*O*-Glc*p* (= astragalin)	*A. eupatoria*	[55]	
	*A. pilosa*	[56]	
	*A. procera*	[36]	
Kaempferol-3-*O*-(6″-Ac)-Glc*p*	*A. pilosa*	[33]	
Kaempferol-3-*O*-[6′-*O*-(*S*)-3-hydroxy-3-methylglutaryl]-Glc*p*	*A. pilosa*	[37]	
Kaempferol-3-*O*-(6″-*p*-CouA)-Glc*p* (= tiliroside)	*A. eupatoria*	[34]	
	*A. pilosa*	[57]	
Kaempferol-7-*O*-Glc*p*	*A. pilosa*	[37]	
Kaempferol-7-*O*-GlcA*p*	*A. pilosa*	[37]	
Kaempferol-3-*O*-Rut (= nicotiflorin)	*A. eupatoria*	[55]	
Kaempferide	*A. eupatoria*	[55]	
Kaempferide-3-*O*-Rha*p*	*A. eupatoria*	[55]	
Quercetin	*A. eupatoria*	[51]	
	*A. pilosa*	[58]	
Quercetin-3-*O*-Glc*p* (= isoquercitrin)	*A. eupatoria*	[36]	
	*A. procera*	[36]	
Quercetin-3-*O*-(6″-*O*-Gall)-Glc*p*	*A. eupatoria*	[36]	
	*A. procera*	[36]	
Quercetin-3-*O*-Gal*p* (= hyperoside)	*A. eupatoria*	[34]	
	*A. pilosa*	[58]	
	*A. procera*	[36]	
Quercetin-3-*O*-Rha*p* (= quercitrin)	*A. eupatoria*	[36]	
	*A. pilosa*	[58]	
	*A. procera*	[36]	
Quercetin-3-*O*-Rut (= rutin)	*A. eupatoria*	[36]	
	*A. pilosa*	[58]	
	*A. procera*	[36]	
Quercetin-3′-*O*-Glc*p*	*A. pilosa*	[37]	
Catechins			
Catechin	*A. eupatoria*	[34]	
	*A. pilosa*	[58]	
Catechin-(5, 6-bc)-4-β-(4″-hydroxyphenyl)-dihydro-2(H)-pyranone	*A. pilosa*	[33]	
Pilosanol A	*A. pilosa*	[49]	[59]
Pilosanol B	*A. pilosa*	[49]	[59]
Pilosanol C	*A. pilosa*	[49]	[59]
Pilosanol N	*A. pilosa*	[60]	
Isopilosanol A	*A. pilosa*	[49]	
Isopilosanol B	*A. pilosa*	[49]	
Isopilosanol C	*A. pilosa*	[49]	
Pilosandin A	*A. pilosa*	[49]	
Pilosandin B	*A. pilosa*	[49]	
Dehydrodicatechin A	*A. pilosa*	[33]	
Proanthocyanidins			
Proanthocyanidin B1	*A. eupatoria*	[34]	
Proanthocyanidin B2	*A. eupatoria*	[34]	
Proanthocyanidin B3	*A. eupatoria*	[34]	
Proanthocyanidin B6	*A. eupatoria*	[34]	
Proanthocyanidin B7	*A. eupatoria*	[34]	
Proanthocyanidin C1	*A. eupatoria*	[34]	
Proanthocyanidin C2	*A. eupatoria*	[34]	
Flavanonols			
Dihydrokaempferol	*A. pilosa*	[47]	
Dihydrokaempferol-3-*O*-Glc*p*	*A. pilosa*	[47]	
(2*R*,3*R*)-Dihydrokaempferol-3-*O*-Glc*p*	*A. pilosa*	[61]	
(2*S*,3*S*)-Dihydrokaempferol-3-*O*-Glc*p*	*A. pilosa*	[61]	
(2*R*,3*S*)-Dihydrokaempferol-3-*O*-Glc*p*	*A. pilosa*	[61]	
(2*S*,3*R*)-Dihydrokaempferol-3-*O*-Glc*p*	*A. pilosa*	[61]	
Dihydroquercetin-3-*O*-Glc*p* (= glucodistylin)	*A. pilosa*	[39]	[62]
(2*R*,3*S*)-Dihydroquercetin-3-*O*-Glc*p*	*A. pilosa*	[61]	
(2*R*,3*R*)-Dihydroquercetin-3-*O*-Glc*p*	*A. pilosa*	[61]	
(2*S*,3*R*)-Dihydroquercetin-3-*O*-Glc*p*	*A. pilosa*	[61]	
(2*S*,3*S*)-Dihydroquercetin-3-*O*-Glc*p*	*A. pilosa*	[61]	
(2*R*,3*R*)-Dihydroquercetin-7-*O*-Glc*p*	*A. pilosa*	[61]	
(2*R*,3*R*)-Dihydroquercetin-4′-*O*-Glc*p*	*A. pilosa*	[61]	
Ellagitannins			
Agritannin (= 1-*O*-Gall-2,3-HHDP-Glc*p*)	*A. eupatoria*	[35]	
	*A. pilosa*	[37]	
Pedunculagin (= 2,3:4,6-Bis-*O*-HHDP-Glc*p*)	*A. pilosa*	[63]	
Agrimoniin [= 1-*O*-{(2″,3′:4′,6′-bis-*O*-HHDP-Glc*p*)-DHDG}-2,3:4,6-bis-*O*-HHDP-Glc*p*]	*A. eupatoria*	[45]	
	*A. pilosa*	[63]	[63]
	*A. procera*	[36]	
Potentillin (= 1-*O*-Gall-2,3:4,6-bis-*O*-HHDP-Glc*p*)	*A. pilosa*	[63]	[64]
Agrimonic acid A (= 1-*O*-DHDG-2,3:4,6-bis-*O*-HHDP-Glc*p*)	*A. pilosa*		[64]
Agrimonic acid B (= 1-*O*-DHDG-2,3:4,6-bis-*O*-HHDP-Glc*p*)	*A. pilosa*		[64]
Sterols			
β-Sitosterol	*A. pilosa*		[44]
Daucosterol	*A. pilosa*		[62]
Triterpenoids			
Oleanolic acid	*A. pilosa*	[65]	
18α-Oleanolic acid	*A. pilosa*	[65]	
Ursolic acid	*A. asiatica*	[32]	
	*A. pilosa*	[66]	
19α-Hydroxyursolic acid	*A. pilosa*	[65]	
Pomolic acid	*A. pilosa*	[66]	
1β-Hydroxy-2-oxopomolic acid	*A. pilosa*	[67]	
2β-Hydroxypomolic acid	*A. pilosa*	[65]	
3-*O*-Acetylpomolic acid	*A. pilosa*	[65]	
Tormentic acid	*A. pilosa*	[66]	[38]
Epitormentic acid	*A. pilosa*	[68]	
Corosolic acid	*A. pilosa*	[66]	
(1*S*,3*R*,17*R*,18*R*,19*R*,20*R*,22*R*)-1,3,19,22-Tetrahydroxy-28-norurs-12-en-2-one	*A. pilosa*	[68]	
1β,2α,3β,19α-Tetrahydroxyurs-12-en-28-oic acid	*A. pilosa*		[41]
1β,2β,3β,19α-Tetrahydroxyurs-12-en-28-oic acid	*A. pilosa*	[68]	[41]
1β,3α,19α-Trihydroxy-2-oxours-12-en-28-oic acid	*A. pilosa*	[65]	
1β,2α,19α-Trihydroxy-3-oxours-12-en-28-oic acid	*A. pilosa*	[65]	
Maslinic acid	*A. pilosa*	[65]	
Rosamultin (= 2α,19α-dihydroxyursolic acid-28-*O*-Glc*p*)	*A. pilosa*	[65]	[41]
Ziyu-glucoside II (= 19α-hydroxyurs-12-enoic acid-3-*O*-Ara*p*)	*A. pilosa*	[65]	
Various groups			
Loliolide	*A. pilosa*	[47]	
Tianshic acid	*A. pilosa*	[33]	
Dihydrodehydrodiconifeyl alcohol-9′-*O*-Glc*p*	*A. pilosa*	[37]	

Abbreviation used: Ac—acetyl; Ara*f*—arabinofuranose; Ara*p*—arabinopyranose; Bu—butyl; DHDG—dehydrodigalloyl; Gall—galloyl; Gal*p*—galactopyranose; Glc*p*—glucopyranose; GlcA*p*—glucuronopyranose; HHDP—hexahydroxydiphenoyl; Me—methyl; *p*-CouA—*p*-coumaroyl; Rha*p*—rhamnopyranose; Rut—rutinose (6-*O*-rhamnopyranosyl-glucopyranose); Xyl*p*—xylopyranose.

**Table 3:** Chromatographic (*t*_R_), mass-spectrometric data (ESI-MS), and seasonal presence/content of compounds **1**–**60** found in the *Agrimonia asiatica* herb.

No	*t*_R_, min	ESI-MS, [M–H]^–^	Group ^a^	Compound ^b^	Content, mg/g Dry Plant Weight ± S.D.		
					May	July	September
**1**	1.38	341	S	Hexosyl-*O*-hexose ^L^	tr.	tr.	tr.
**2**	1.52	331	GT	1-*O*-Galloyl-glucose ^S^	tr.	n.f.	n.f.
**3**	1.88	331	GT	*O*-Galloyl-glucose ^L^	tr.	n.f	n.f.
**4**	1.94	331	GT	*O*-Galloyl-glucose ^L^	tr.	tr.	n.f.
**5**	2.11	331	GT	*O*-Galloyl-glucose ^L^	tr.	n.f.	n.f.
**6**	2.36	183	V	2-Pyrone-4,6-dicarboxylic acid ^S^	1.42 ± 0.03	2.05 ± 0.04	2.11 ± 0.03
**7**	2.67	783	ET	Pedunculagin ^S^	tr.	tr.	tr.
**8**	2.91	353	HC	4-*O*-Caffeoylquinic acid ^S^	15.63 ± 0.32	18.59 ± 0.37	16.37 ± 0.31
**9**	3.22	783	ET	Casuariin ^S^	tr.	tr.	tr.
**10**	4.01	577	P	Procyanidin B_1_^S^	tr.	tr.	tr.
**11**	4.26	785	ET	Tellimagrandin I_1_^S^	tr.	tr.	n.f.
**12**	4.51	577	P	Procyanidin B3 ^S^	tr.	tr.	tr.
**13**	4.76	289	C	Catechin ^S^	20.19 ± 0.41	22.27 ± 0.45	11.63 ± 0.19
**14**	5.31	353	HC	5-*O*-Caffeoylquinic acid ^S^	tr.	tr.	tr.
**15**	5.51	305	C	Epigallocatechin ^S^	27.02 ± 0.54	65.15 ± 1.30	36.11 ± 0.72
**16**	6.09	577	P	Procyanidin B2 ^S^	tr.	tr.	tr.
**17**	6.33	447	V	Ellagic acid-*O*-methyl ester-*O*-pentoside ^L^	n.f.	tr.	tr.
**18**	6.59	937	ET	Tri-*O*-galloyl-*O*-hexahydroxydiphenoyl-glucose ^L^	tr.	tr.	n.f.
**19**	6.92	785	ET	Tellimagrandin I_2_^S^	tr.	tr.	n.f.
**20**	7.54	577	P	Procyanidin B (dimer) ^L^	tr.	tr.	tr.
**21**	8.03	783	ET	Bis-*O*-hexahydroxydiphenoyl-glucose ^L^	tr.	tr.	n.f.
**22**	8.20	785	ET	Di-*O*-galloyl-*O*-hexahydroxydiphenoyl-glucose ^L^	tr.	tr.	n.f.
**23**	8.49	289	C	Epicatechin ^S^	tr.	tr.	tr.
**24**	8.71	337	ET	Hexahydroxydiphenic acid ^L^	tr.	tr.	n.f.
**25**	9.08	785	ET	Di-*O*-galloyl-*O*-hexahydroxydiphenoyl-glucose ^L^	tr.	tr.	n.f.
**26**	9.46	515	HC	1,3-Di-*O*-caffeoyquinic acid ^S^	tr.	tr.	tr.
**27**	9.54	783	ET	Bis-*O*-hexahydroxydiphenoyl-glucose ^L^	tr.	tr.	n.f.
**28**	9.69	463	F	6-Hydroxyluteolin-7-*O*-Glc ^S^	n.f.	tr.	n.f.
**29**	10.02	783	ET	Bis-*O*-hexahydroxydiphenoyl-glucose ^L^	tr.	tr.	n.f.
**30**	10.27	577	P	Procyanidin B (dimer) ^L^	n.f.	tr.	tr.
**31**	10.42	937	ET	Tellimagrandin II_1_^S^	tr.	tr.	n.f.
**32**	10.71	935	ET	Potentillin ^S^	tr.	tr.	tr.
**33**	11.01	1103	ET	Agrimonic acid A ^S^	tr.	tr.	n.f.
**34**	11.12	1871	ET	Gemin A ^S^	tr.	tr.	tr.
**35**	11.48	1103	ET	Agrimonic acid B ^S^	tr.	tr.	n.f.
**36**	11.63	1869	ET	Agrimoniin ^S^	82.59 ± 1.67	114.18 ±2.37	27.32 ± 0.54
**37**	11.97	441	C	Epicatechin gallate ^S^	tr.	tr.	tr.
**38**	12.27	301	V	Ellagic acid ^S^	3.60 ± 0.06	5.31 ± 0.12	34.62 ± 0.69
**39**	12.51	609	F	Quercetin-3-*O*-(6″-*O*-rhamnosyl)-glucoside ^S^	8.06 ± 0.17	16.32 ± 0.31	17.83 ± 0.35
**40**	12.98	937	ET	Tellimagrandin II_2_^S^	tr.	tr.	n.f.
**41**	13.41	463	F	Quercetin-3-*O*-glucoside ^S^	9.53 ± 0.17	29.80 ± 0.61	24.18 ± 0.48
**42**	13.50	477	F	Quercetin-3-*O*-glucuronide ^S^	tr.	tr.	tr.
**43**	13.89	447	F	Luteolin-7-*O*-glucoside ^S^	tr.	tr.	tr.
**44**	14.21	461	F	Luteolin-7-*O*-glucuronide ^S^	2.16 ± 0.04	4.21 ± 0.09	4.69 ± 0.08
**45**	15.20	549	F	Quercetin-3-*O*-(6″-*O*-malonyl)-glucoside ^S^	6.37 ± 0.12	4.14 ± 0.08	0.92 ± 0.02
**46**	15.67	515	HC	3,5-Di-*O*-caffeoyquinic acid ^S^	2.40 ± 0.04	2.59 ± 0.05	0.31 ± 0.01
**47**	16.01	593	F	Kaempferol-3-*O*-(6″-*O*-rhamnosyl)-glucoside ^S^	7.53 ± 0.15	9.27 ± 0.19	11.36 ± 0.21
**48**	16.20	447	F	Quercetin-3-*O*-rhamnoside ^S^	tr.	tr.	tr.
**49**	16.51	447	F	Kaempferol-3-*O*-glucoside ^S^	tr.	tr.	tr.
**50**	16.55	431	F	Apigenin-7-*O*-glucoside ^S^	tr.	0.75 ± 0.02	0.82 ± 0.02
**51**	17.08	445	F	Apigenin-7-*O*-glucuronide ^S^	22.18 ± 0.44	47.22 ± 0.94	56.14 ± 1.14
**52**	17.27	533	F	Kaempferol-*O*-malonyl-*O*-hexoside ^L^	tr.	tr.	tr.
**53**	17.91	533	F	Luteolin-3-*O*-(6″-*O*-malonyl)-glucoside ^S^	2.10 ± 0.04	0.85 ± 0.02	n.f.
**54**	18.23	431	F	Kaempferol-3-*O*-rhamnoside ^S^	0.21 ± 0.00	0.59 ± 0.01	0.73 ± 0.01
**55**	19.43	609	F	Quercetin-3-*O*-(6″-*O*-*p*-coumaroyl)-glucoside ^S^	tr.	tr.	tr.
**56**	20.01	593	F	Kaempferol-3-*O*-(6″-*O*-*p*-coumaroyl)-glucoside ^S^	tr.	tr.	tr.
**57**	20.87	329	V	Ellagic acid di-*O*-methyl ester ^L^	tr.	tr.	tr.
**58**	21.63	593	F	Luteolin-7-*O*-(6″-*O*-*p*-coumaroyl)-glucoside ^S^	tr.	tr.	n.f.
**59**	22.24	517	F	Apigenin-7-*O*-(6″-*O*-*p*-malonyl)-glucoside ^S^	tr.	tr.	tr.
**60**	24.43	577	F	Apigenin-7-*O*-(6″-*O*-*p*-coumaroyl)-glucoside ^S^	tr.	tr.	tr.

^a^ Chemical group of compound: C—catechins, ET—ellagitannins, F—flavones/flavonols, GT—gallotannins, HC—hydroxycinnamates, P—procyanidins, S—sugars, V—various compounds. ^b^ The identification of compounds was realized on comparison of retention time, UV and MS spectral data with a reference standard (^S^) or interpretation of UV and MS spectral data and comparison with literature data (^L^). tr.—trace, n.f.—not found.

**Table 4:** Phenolic content in agrimonia tea infusions and decoctions (mg/100 mL ± S.D.) and their α-glucosidase inhibiting potential (αGIP, IC_50_, μg/mL).

Compound	Cold Infusion (20 °C)	Warm Infusion (50 °C)	Hot Infusion (80 °C)	Boiling Infusion (100 °C)	Decoction 15 min	Decoction 30 min
Ellagitannins						
Agrimoniin	30.32 ± 0.60	34.14 ± 0.68	36.73 ± 0.73	26.67 ± 0.53	4.75 ± 0.09	traces
Casuariin	1.12 ± 0.02	1.29 ± 0.03	1.47 ± 0.03	0.88 ± 0.02	traces	traces
Subtotal ellagitannins	31.44	35.43	38.20	27.55	4.75	traces
Catechins						
Catechin	1.70 ± 0.03	1.73 ± 0.04	2.54 ± 0.05	3.47 ± 0.07	5.01 ± 0.10	4.83 ± 0.09
Epigallocatechin	4.45 ± 0.09	5.44 ± 0.11	7.37 ± 0.14	10.26 ± 0.20	17.96 ± 0.35	18.26 ± 0.36
Subtotal catechins	6.15	7.17	9.91	13.74	22.97	23.09
Hydroxycinnamates						
4-*O*-Caffeoylquinic acid	3.51 ± 0.06	4.16 ± 0.08	5.01 ± 0.10	6.06 ± 0.12	5.99 ± 0.14	5.84 ± 0.14
3,5-Di-*O*-caffeoyquinic acid	0.35 ± 0.01	0.44 ± 0.01	0.55 ± 0.01	0.59 ± 0.01	0.53 ± 0.02	0.51 ± 0.02
Subtotal hydroxycinnamates	3.86	4.60	5.56	6.65	6.52	6.35
Flavones/Flavonols						
Quercetin-3-*O*-(6″-*O*-rhamnosyl)-glucoside	2.94 ± 0.06	3.42 ± 0.07	4.01 ± 0.08	7.47 ± 0.15	7.83 ± 0.16	7.94 ± 0.18
Quercetin-3-*O*-glucoside	4.23 ± 0.08	4.61 ± 0.09	6.44 ± 0.12	6.76 ± 0.14	7.66 ± 0.15	7.73 ± 0.16
Quercetin-3-*O*-(6″-*O*-malonyl)-glucoside	0.64 ± 0.02	0.65 ± 0.02	1.12 ± 0.02	traces	traces	traces
Kaempferol-3-*O*-(6″-*O*-rhamnosyl)-glucoside	1.70 ± 0.03	1.83 ± 0.03	2.08 ± 0.04	2.24 ± 0.04	2.35 ± 0.05	2.57 ± 0.05
Kaempferol-3-*O*-rhamnoside	traces	traces	0.09 ± 0.00	0.27 ± 0.00	0.26 ± 0.00	0.26 ± 0.00
Luteolin-7-*O*-glucuronide	0.77 ± 0.02	1.46 ± 0.03	1.52 ± 0.03	2.04 ± 0.04	2.06 ± 0.04	2.11 ± 0.04
Luteolin-3-*O*-(6″-*O*-malonyl)-glucoside	0.26 ± 0.00	0.34 ± 0.01	0.40 ± 0.01	traces	traces	traces
Apigenin-7-*O*-glucoside	traces	0.08 ± 0.00	0.15 ± 0.00	0.17 ± 0.00	0.17 ± 0.00	0.16 ± 0.00
Apigenin-7-*O*-glucuronide	8.47 ± 0.16	9.97 ± 0.19	11.06 ± 0.23	11.91 ± 0.23	12.96 ± 0.25	13.27 ± 0.27
Subtotal flavones/flavonols	19.01	22.36	26.87	30.86	33.29	33.78
Various Phenolics						
2-Pyrone-4,6-dicarboxylic acid	0.32 ± 0.01	0.51 ± 0.01	0.81 ± 0.02	1.15 ± 0.02	1.87 ± 0.04	1.92 ± 0.04
Ellagic acid	1.25 ± 0.02	1.25 ± 0.02	1.41 ± 0.03	3.65±0.07	20.79 ± 0.42	21.14 ± 0.43
Subtotal various phenolics	1.57	1.76	2.22	4.80	22.66	23.06
Total phenolics	62.03	71.32	82.76	83.59	90.19	86.54
αGIP, IC_50_	89.14	86.89	75.33	96.11	141.17	159.14

## Supplementary Materials

The following are available online at https://www.mdpi.com/2304-8158/9/10/1348/s1, Table S1: Description of 85 Rosaceous plants used in the study, Table S2: Mass spectrometric parameters of compounds 1–60.
