# Supplementary material for: Phenolome of Asian Agrimony Tea (Agrimonia asiatica Juz., Rosaceae): LC-MS Profile, α-Glucosidase Inhibitory Potential and Stability
Source: Foods. 2020 Sep 23;9(10):1348. doi: 10.3390/foods9101348 (PMC7598702; doi:10.3390/foods9101348)
Supplement: Supplementary file 1 [file foods-09-01348-s001.pdf]

foods

Supplementary Materials

Phenolome of Asian Agrimony Tea (*Agrimonia asiatica* Juz., Rosaceae): LC-MS Profile, α-Glucosidase Inhibitory Potential and Stability

Nina I. Kashchenko * and Daniil N. Olennikov

Laboratory of Medical and Biological Research, Institute of General and Experimental Biology, Siberian Division, Russian Academy of Science, 6 Sakh'yanovoy Street, Ulan-Ude 670047, Russia; olennikovdn@mail.ru (D.N.O.)

* Correspondence: ninkk@mail.ru; Tel.: +79-0216-00-627 (D.N.O.)

Received: date; Accepted: date; Published: date

Content

Table S1. Description of 85 Rosaceous plants used in study.

Table S2. Mass spectrometric parameters of compounds **1**–**60**.

Table S1. Description of 85 Rosaceous plants used in study.

Species	Collection place	Coordinates	Collection date
Agrimonia pilosa Ledeb.	Republic Buryatia, Kyren (Tunkinsky District)	51°41′15.4501′′ N, 102°8′21.8127′′ E, 947 m	21.VII.2019
Alchemilla anisopoda Juz.	Republic Buryatia, Kabansk (Kabansky District)	52°2′54.5243′′ N, 106°37′34.5456′′ E, 461 m	23.VII.2019
A. flavescens Buser	Republic Buryatia, Kolobki (Ivolginsky District)	51°38′53.0286′′ N, 107°23′54.2239′′ E, 506 m	02.VII.2019
A. subcrenata Buser	Republic Buryatia, Severobaykalsk	55°38′56.8229′′ N, 109°20′28.5324′′ E, 582 m	02.VIII.2019
Armeniaca sibirica (L.) Lam.	Republic Buryatia, Podlopatki (Mukhorshibirsky District)	50°54′50.8598′′ N, 107°6′9.5972′′ E, 647 m	29.V.2018
Chamaerhodos erecta (L.) Bunge	Republic Buryatia, Zhemchug (Tunkinsky District)	51°41′51.0445′′ N, 102°28′14.9261′′ E, 722 m	17.VII.2019
C. grandiflora (Pall. ex Schult.) Bunge	Republic Buryatia, Tankhoy (Kabansky District)	51°32′59.4879′′ N, 105°7′15.1616′′ E, 504 m	15.VII.2018
Cotoneaster lucidus Schltdl.	Republic Buryatia, Zaigrayevo (Zaigraevsky District)	51°51′6.8316′′ N, 108°18′36.9388′′ E, 639 m	11.VII.2019
Cotoneaster melanocarpus Fisch. ex Blytt	Republic Buryatia, Mukhorshibir (Mukhorshibirsky district)	51°1′54.8212′′ N, 107°54′10.4700′′ E, 1003 m	18.VII.2019
C. mongolicus Pojark.	Republic Buryatia, Zun-Murino (Tunkinsky District)	51°44′27.2468′′ N, 102°53′40.4121′′ E, 724 m	23.VII.2018
C. neopopovii Czerep.	Republic Buryatia, Baunt (Bauntovsky District)	55°15′37.2172′′ N, 113°8′57.2040′′ E, 1096 m	01.VIII.2019
C. tjuliniae Pojark. ex Peschkova	Republic Buryatia, Kolobki (Ivolginsky District)	51°38′53.0286′′ N, 107°23′54.2239′′ E, 506 m	02.VII.2019
C. uniflorus Bunge	Republic Buryatia, Zhemchug (Tunkinsky District)	51°41′51.0445′′ N, 102°28′14.9261′′ E, 722 m	17.VII.2019
Crataegus dahurica Koehne ex C.K.Schneid.	Republic Buryatia, Podlopatki (Mukhorshibirsky District)	50°54′50.8598′′ N, 107°6′9.5972′′ E, 647 m	29.V.2018
C. maximowiczii C.K.Schneid.	Republic Buryatia, Zun-Murino (Tunkinsky District)	51°44′27.2468′′ N, 102°53′40.4121′′ E, 724 m	23.VII.2018

Table S1. Continuation

Species	Collection place	Coordinates	Collection date
C. sanguinea Pall.	Republic Buryatia, Mukhorshibir (Mukhorshibirsky district)	51°1′54.8212′′ N, 107°54′10.4700′′ E, 1003 m	18.VII.2019
Dasiphora fruticosa (L.) Rydb.	Republic Buryatia, Tankhoy (Kabansky District)	51°32′59.4879′′ N, 105°7′15.1616′′ E, 504 m	15.VII.2018
Dryas grandis Juz.	Republic Buryatia, Kyren (Tunkinsky District)	51°41′15.4501′′ N, 102°8′21.8127′′ E, 947 m	21.VII.2019
D. incisa Juz.	Republic Buryatia, Kyren (Tunkinsky District)	51°41′15.4501′′ N, 102°8′21.8127′′ E, 947 m	21.VII.2019
D. integrifolia subsp. crenulata (Juz.) Kozhevn.	Republic Buryatia, Kyren (Tunkinsky District)	51°41′15.4501′′ N, 102°8′21.8127′′ E, 947 m	21.VII.2019
D. oxyodonta Juz.	Republic Buryatia, Kyren (Tunkinsky District)	51°41′15.4501′′ N, 102°8′21.8127′′ E, 947 m	21.VII.2019
D. sumneviczii Serg.	Republic Buryatia, Baunt (Bauntovsky District)	55°15′37.2172′′ N, 113°8′57.2040′′ E, 1096 m	01.VIII.2019
Dasiphora parvifolia (Fisch.) Juz.	Republic Buryatia, Kolobki (Ivolginsky District)	51°38′53.0286′′ N, 107°23′54.2239′′ E, 506 m	02.VII.2019
Fragaria vesca L.	Republic Buryatia, Mukhorshibir (Mukhorshibirsky district)	51°1′54.8212′′ N, 107°54′10.4700′′ E, 1003 m	18.VII.2019
F. orientalis Losinsk.	Republic Buryatia, Mukhorshibir (Mukhorshibirsky district)	51°1′54.8212′′ N, 107°54′10.4700′′ E, 1003 m	18.VII.2019
Geum aleppicum Jacq.	Republic Buryatia, Onokhoy (Zaigraevsky District)	51°55′10.1889′′ N, 108°3′26.4983′′ E, 544 m	03.VII.2019
G. rivale L.	Republic Buryatia, Zun-Murino (Tunkinsky District)	51°44′27.2468′′ N, 102°53′40.4121′′ E, 724 m	23.VII.2018
Malus baccata (L.) Borkh.	Republic Buryatia, Podlopatki (Mukhorshibirsky District)	50°54′50.8598′′ N, 107°6′9.5972′′ E, 647 m	29.V.2018
Padus avium Mill.	Republic Buryatia, Zaigrayevo (Zaigraevsky District)	51°51′6.8316′′ N, 108°18′36.9388′′ E, 639 m	11.VI.2019
Potentilla acaulis L.	Republic Buryatia, Tankhoy (Kabansky District)	51°32′59.4879′′ N, 105°7′15.1616′′ E, 504 m	15.VII.2018
P. acervata Soják	Republic Buryatia, Baunt (Bauntovsky District)	55°15′37.2172′′ N, 113°8′57.2040′′ E, 1096 m	01.VIII.2019
P. adenotricha Vodop.	Republic Buryatia, Onokhoy (Zaigraevsky District)	51°55′10.1889′′ N, 108°3′26.4983′′ E, 544 m	03.VII.2019
P. anserina L.	Republic Buryatia, Podlopatki (Mukhorshibirsky District)	50°54′50.8598′′ N, 107°6′9.5972′′ E, 647 m	29.V.2018

Table S1. Continuation

Species	Collection place	Coordinates	Collection date
P. arenosa (Turcz.) Juz.	Republic Buryatia, Mukhorshibir (Mukhorshibirsky district)	51°1′54.8212′′ N, 107°54′10.4700′′ E, 1003 m	18.VII.2019
P. argentea L.	Republic Buryatia, Zun-Murino (Tunkinsky District)	51°44′27.2468′′ N, 102°53′40.4121′′ E, 724 m	23.VII.2018
P. asperrima Turcz.	Republic Buryatia, Kyakhta (Kyakhtinsky District)	50°22′56.5082′′ N, 106°26′58.9522′′ E, 853 m	13.VII.2019
P. biflora Willd. ex Schltdl.	Republic Buryatia, Podlopatki (Mukhorshibirsky District)	50°54′50.8598′′ N, 107°6′9.5972′′ E, 647 m	29.V.2018
P. chrysantha Trevir.	Republic Buryatia, Onokhoy (Zaigraevsky District)	51°55′10.1889′′ N, 108°3′26.4983′′ E, 544 m	03.VII.2019
P. conferta Bunge	Republic Buryatia, Zhemchug (Tunkinsky District)	51°41′51.0445′′ N, 102°28′14.9261′′ E, 722 m	17.VII.2019
P. crantzii (Crantz) Beck ex Fritsch	Republic Buryatia, Zaigrayevo (Zaigraevsky District)	51°51′6.8316′′ N, 108°18′36.9388′′ E, 639 m	11.VII.2019
P. desertorum Bunge	Republic Buryatia, Kabansk (Kabansky District)	52°2′54.5243′′ N, 106°37′34.5456′′ E, 461 m	23.VII.2019
P. elegans Cham. & Schltdl.	Republic Buryatia, Tankhoy (Kabansky District)	51°32′59.4879′′ N, 105°7′15.1616′′ E, 504 m	15.VII.2018
P. evestita Th.Wolf	Republic Buryatia, Mukhorshibir (Mukhorshibirsky district)	51°1′54.8212′′ N, 107°54′10.4700′′ E, 1003 m	18.VII.2019
P. flagellaris Willd. ex Schltdl.	Republic Buryatia, Kyakhta (Kyakhtinsky District)	50°22′56.5082′′ N, 106°26′58.9522′′ E, 853 m	13.VII.2019
P. fragarioides L.	Republic Buryatia, Kolobki (Ivolginsky District)	51°38′53.0286′′ N, 107°23′54.2239′′ E, 506 m	02.VII.2019
P. kryloviana Th.Wolf	Republic Buryatia, Kyakhta (Kyakhtinsky District)	50°22′56.5082′′ N, 106°26′58.9522′′ E, 853 m	13.VII.2019
P. leucophylla Pall.	Republic Buryatia, Zaigrayevo (Zaigraevsky District)	51°51′6.8316′′ N, 108°18′36.9388′′ E, 639 m	11.VII.2019
P. longifolia Willd. ex Schltdl.	Republic Buryatia, Baunt (Bauntovsky District)	55°15′37.2172′′ N, 113°8′57.2040′′ E, 1096 m	01.VIII.2019
P. multifida L.	Republic Buryatia, Onokhoy (Zaigraevsky District)	51°55′10.1889′′ N, 108°3′26.4983′′ E, 544 m	03.VII.2019

Table S1. Continuation

Species	Collection place	Coordinates	Collection date
P. mujensis Kurbatski	Republic Buryatia, Severobaykalsk	55°38′56.8229′′ N, 109°20′28.5324′′ E, 582 m	02.VIII.2019
P. nivea L.	Republic Buryatia, Zun-Murino (Tunkinsky District)	51°44′27.2468′′ N, 102°53′40.4121′′ E, 724 m	23.VII.2018
P. norvegica L.	Republic Buryatia, Mukhorshibir (Mukhorshibirsky district)	51°1′54.8212′′ N, 107°54′10.4700′′ E, 1003 m	18.VII.2019
P. nudicaulis Willd. ex Schltdl.	Republic Buryatia, Zhemchug (Tunkinsky District)	51°41′51.0445′′ N, 102°28′14.9261′′ E, 722 m	17.VII.2019
P. ozjorensis Peschkova	Republic Buryatia, Kabansk (Kabansky District)	52°2′54.5243′′ N, 106°37′34.5456′′ E, 461 m	23.VII.2019
P. sanguisorba Willd. ex Schltdl.	Republic Buryatia, Zaigrayevo (Zaigraevsky District)	51°51′6.8316′′ N, 108°18′36.9388′′ E, 639 m	11.VII.2019
P. saposhnikovii Kurbatski	Republic Buryatia, Baunt (Bauntovsky District)	55°15′37.2172′′ N, 113°8′57.2040′′ E, 1096 m	01.VIII.2019
P. sericea L.	Republic Buryatia, Kolobki (Ivolginsky District)	51°38′53.0286′′ N, 107°23′54.2239′′ E, 506 m	02.VII.2019
P. stipularis L.	Republic Buryatia, Onokhoy (Zaigraevsky District)	51°55′10.1889′′ N, 108°3′26.4983′′ E, 544 m	03.VII.2019
P. tanacetifolia Willd. ex Schltdl.	Republic Buryatia, Kabansk (Kabansky District)	52°2′54.5243′′ N, 106°37′34.5456′′ E, 461 m	23.VII.2019
P. tergemina Soják	Republic Buryatia, Kolobki (Ivolginsky District)	51°38′53.0286′′ N, 107°23′54.2239′′ E, 506 m	02.VII.2019
P. verticillaris Stephan ex Willd.	Republic Buryatia, Kyakhta (Kyakhtinsky District)	50°22′56.5082′′ N, 106°26′58.9522′′ E, 853 m	13.VII.2019
Prunus pedunculata (Pall.) Maxim.	Republic Buryatia, Zhemchug (Tunkinsky District)	51°41′51.0445′′ N, 102°28′14.9261′′ E, 722 m	17.VII.2019
Rosa acicularis Lindl.	Republic Buryatia, Mukhorshibir (Mukhorshibirsky district)	51°1′54.8212′′ N, 107°54′10.4700′′ E, 1003 m	18.VII.2019
R. davurica Pall.	Republic Buryatia, Zaigrayevo (Zaigraevsky District)	51°51′6.8316′′ N, 108°18′36.9388′′ E, 639 m	11.VII.2019
Rubus arcticus L.	Republic Buryatia, Tankhoy (Kabansky District)	51°32′59.4879′′ N, 105°7′15.1616′′ E, 504 m	15.VII.2018

Table S1. Continuation

Species	Collection place	Coordinates	Collection date
Rubus chamaemorus L.	Republic Buryatia, Zun-Murino (Tunkinsky District)	51°44′27.2468′′ N, 102°53′40.4121′′ E, 724 m	23.VII.2018
R. humulifolius C.A. Mey.	Republic Buryatia, Onokhoy (Zaigraevsky District)	51°55′10.1889′′ N, 108°3′26.4983′′ E, 544 m	03.VII.2019
R. matsumuranus H. Lev. & Vaniot	Republic Buryatia, Tankhoy (Kabansky District)	51°32′59.4879′′ N, 105°7′15.1616′′ E, 504 m	15.VII.2018
R. saxatilis L.	Republic Buryatia, Mukhorshibir (Mukhorshibirsky district)	51°1′54.8212′′ N, 107°54′10.4700′′ E, 1003 m	18.VII.2019
Sanguisorba alpina Bunge	Republic Buryatia, Severobaykalsk	55°38′56.8229′′ N, 109°20′28.5324′′ E, 582 m	02.VIII.2019
S. officinalis L.	Republic Buryatia, Kabansk (Kabansky District)	52°2′54.5243′′ N, 106°37′34.5456′′ E, 461 m	23.VII.2019
Sibbaldia adpressa Bunge	Republic Buryatia, Zun-Murino (Tunkinsky District)	51°44′27.2468′′ N, 102°53′40.4121′′ E, 724 m	23.VII.2018
S. procumbens L.	Republic Buryatia, Kyakhta (Kyakhtinsky District)	50°22′56.5082′′ N, 106°26′58.9522′′ E, 853 m	13.VII.2019
Sibbaldianthe bifurca (L.) Kurtto & T.Erikss.	Republic Buryatia, Zhemchug (Tunkinsky District)	51°41′51.0445′′ N, 102°28′14.9261′′ E, 722 m	17.VII.2019
S. bifurca subsp. orientalis (Juz.) Kurtto & T.Erikss.	Republic Buryatia, Kolobki (Ivolginsky District)	51°38′53.0286′′ N, 107°23′54.2239′′ E, 506 m	02.VII.2019
Sorbaria pallasii (G. Don fil.) Pojark.	Republic Buryatia, Podlopatki (Mukhorshibirsky District)	50°54′50.8598′′ N, 107°6′9.5972′′ E, 647 m	29.V.2018
S. sorbifolia (L.) A. Braun	Republic Buryatia, Severobaykalsk	55°38′56.8229′′ N, 109°20′28.5324′′ E, 582 m	02.VIII.2019
Sorbus sibirica Hedl.	Republic Buryatia, Podlopatki (Mukhorshibirsky District)	50°54′50.8598′′ N, 107°6′9.5972′′ E, 647 m	29.V.2018
Spiraea alpina Pall.	Republic Buryatia, Zhemchug (Tunkinsky District)	51°41′51.0445′′ N, 102°28′14.9261′′ E, 722 m	17.VII.2019
S. aquilegifolia Pall.	Republic Buryatia, Baunt (Bauntovsky District)	55°15′37.2172′′ N, 113°8′57.2040′′ E, 1096 m	01.VIII.2019
S. dahurica (Rupr.) Maxim.	Republic Buryatia, Podlopatki (Mukhorshibirsky District)	50°54′50.8598′′ N, 107°6′9.5972′′ E, 647 m	29.V.2018
S. flexuosa Fisch. ex Cambess.	Republic Buryatia, Kolobki (Ivolginsky District)	51°38′53.0286′′ N, 107°23′54.2239′′ E, 506 m	02.VII.2019

Table S1. Continuation

**Species**	**Collection place**	**Coordinates**	**Collection date**
S. media Schmidt	Republic Buryatia, Mukhorshibir (Mukhorshibirsky district)	51°1′54.8212′′ N, 107°54′10.4700′′ E, 1003 m	18.VII.2019
S. salicifolia L.	Republic Buryatia, Kabansk (Kabansky District)	52°2′54.5243′′ N, 106°37′34.5456′′ E, 461 m	23.VII.2019

Table S2. Mass spectrometric parameters of compounds **1–60**.

No	ESI-MS, [M–H]$^-$, *m/z*	ESI-MS, MS/MS, *m/z*	Parent ion, *m/z*	Daughter ion, *m/z*	Declustering potential, V	Collision energy, V	Compound
**1**	341	179	341	179	-35	-5	Hexosyl-*O*-hexose
**2**	331	169	331	169	-47	-15	1-*O*-Galloyl-glucose
**3**	331	169	331	169	-50	-15	*O*-Galloyl-glucose
**4**	331	169	331	169	-40	-15	*O*-Galloyl-glucose
**5**	331	169	331	169	-45	-15	*O*-Galloyl-glucose
**6**	183	139	183	139	-65	-20	2-Pyrone-4,6-dicarboxylic acid
**7**	783	633, 481, 301	783	301	-72	-15	Pedunculagin
**8**	353	191, 179, 173, 135	353	191	-30	-10	4-*O*-Caffeoylquinic acid
**9**	783	621, 471, 301	783	621	-55	-15	Casuariin
**10**	577	305, 289	577	289	-85	-30	Procyanidin B1
**11**	785	623, 473, 301	785	623	-75	-20	Tellimagrandin I_1
**12**	577	305, 289	577	289	-80	-35	Procyanidin B3
**13**	289	125	289	125	-94	-25	(+)-Catechin
**14**	353	191, 165	353	191	-30	-10	5-*O*-Caffeoylquinic acid
**15**	305	155, 125	305	125	-55	-20	(-)Epigallocatechin
**16**	577	289	577	289	-80	-30	Procyanidin B2
**17**	447	315, 301	447	315	-105	-35	Ellagic acid-*O*-methyl ester-*O*-pentoside
**18**	937	775, 625, 475	937	775	-85	-20	Tri-*O*-galloyl-*O*-hexahydroxy diphenoyl-glucose
**19**	785	623, 473, 301	785	623	-75	-20	Tellimagrandin I_2
**20**	577	305, 289	577	289	-85	-20	Procyanidin dimer
**21**	783	621, 391	783	621	-87	-25	Bis-*O*-hexahydroxydiphenoyl-glucose
**22**	785	623, 321, 301	785	623	-85	-20	Di-*O*-galloyl-*O*-hexahydroxy diphenoyl-glucose
**23**	289	125	289	125	-94	-20	(-)-Epicatechin
**24**	337	301, 275, 249	337	301	-102	-30	Hexahydroxydiphenic acid
**25**	785	623, 321, 301	785	623	-82	-15	Di-*O*-galloyl-*O*-hexahydroxy diphenoyl-glucose
**26**	515	353, 191, 179	515	191	-35	-10	1,3-Di-*O*-caffeoyquinic acid
**27**	783	621, 391	783	621	-75	-25	Bis-*O*-hexahydroxydiphenoyl-glucose
**28**	463	301	463	301	-72	-30	6-Hydroxyluteolin-7-*O*-Glc
**29**	783	621, 391	783	621	-79	-25	Bis-*O*-hexahydroxydiphenoyl-glucose
**30**	577	289	577	289	-80	-30	Procyanidin dimer
**31**	937	785, 623, 473, 301	937	785	-87	-20	Tellimagrandin II_1
**32**	935	633, 463, 301	935	633	-80	-20	Potentillin
**33**	1103	801, 783, 499, 481, 319, 301	1103	801	-75	-25	Agrimonic acid A
**34**	1871	1263, 1083, 933, 781, 301	1871	933	-95	-20	Gemin A
**35**	1103	801, 783, 499, 481, 319, 301	1103	801	-80	-25	Agrimonic acid B
**36**	1869	1567, 1265, 1085, 935, 783, 633, 481, 301	1869	935	-85	-25	Agrimoniin
**37**	441	291	441	291	-64	-20	(-)-Epicatechin gallate
**38**	301	285	301	285	-120	-40	Ellagic acid
**39**	609	463, 301	609	301	-106	-30	Quercetin-3-*O*-(6′′-*O*-rhamnosyl) -glucoside
**40**	937	785, 623, 473, 301	937	785	-85	-25	Tellimagrandin II_2
**41**	463	301	463	301	-100	-30	Quercetin-3-*O*-glucoside
**42**	477	301	477	301	-105	-30	Quercetin-3-*O*-glucuronide
**43**	447	285	447	285	-110	-35	Luteolin-7-*O*-glucoside
**44**	461	285	461	285	-110	-35	Luteolin-7-*O*-glucuronide
**45**	549	301	549	301	-95	-30	Quercetin-3-*O*-(6′′-*O*-malonyl)-glucoside
**46**	515	353, 191, 179	515	191	-40	-15	3,5-Di-*O*-caffeoyquinic acid
**47**	593	447, 285	593	285			Kaempferol-3-*O*-(6′′-*O*-rhamno-syl)-glucoside
**48**	447	301	447	301	-105	-30	Quercetin-3-*O*-rhamnoside
**49**	447	285	447	285	-110	-35	Kaempferol-3-*O*-glucoside
**50**	431	269	431	269	-105	-30	Apigenin-7-*O*-glucoside
**51**	445	269	445	269	-110	-35	Apigenin-7-*O*-glucuronide
**52**	533	285	533	285	-106	-30	Kaempferol-*O*-malonyl-*O*-hexoside

Table S2. Continuation

No	ESI-MS, [M–H]⁻, *m*/*z*	ESI-MS, MS/MS, *m*/*z*	Parent ion, *m*/*z*	Daughter ion, *m*/*z*	Declustering potential, V	Collision energy, V	Compound
**53**	533	285	533	285	-115	-30	Luteolin-3-*O*-(6′′-*O*-malonyl)-glucoside
**54**	431	285	431	285	-115	-35	Kaempferol-3-*O*-rhamnoside
**55**	609	301	609	301	-104	-30	Quercetin-3-*O*-(6′′-*O*-*p*-coumaroyl)-glucoside
**56**	593	285	593	285	-110	-30	Kaempferol-3-*O*-(6′′-*O*-*p*-coumaroyl)-glucoside
**57**	329	315, 301	329	315	-130	-40	Ellagic acid di-*O*-methyl ester
**58**	593	285	593	285	-115	-35	Luteolin-7-*O*-(6′′-*O*-*p*-coumaroyl)-glucoside
**59**	517	269	517	269	-120	-35	Apigenin-7-*O*-(6′′-*O*-*p*-malonyl)-glucoside
**60**	577	269	577	269	-120	-35	Apigenin-7-*O*-(6′′-*O*-*p*-coumaroyl)-glucoside
